# Raman spectroscopy as a tool for monitoring mesoscale continuous-flow organic synthesis: Equipment interface and assessment in four medicinally-relevant reactions

**DOI:** 10.3762/bjoc.9.215

**Published:** 2013-09-11

**Authors:** Trevor A Hamlin, Nicholas E Leadbeater

**Affiliations:** 1 Department of Chemistry, University of Connecticut, 55 North Eagleville Road, Storrs, CT 06269, USA

**Keywords:** flow processing, Raman spectroscopy, reaction monitoring, α,β-unsaturated carbonyl

## Abstract

An apparatus is reported for real-time Raman monitoring of reactions performed using continuous-flow processing. Its capability is assessed by studying four reactions, all involving formation of products bearing α,β-unsaturated carbonyl moieties; synthesis of 3-acetylcoumarin, Knoevenagel and Claisen–Schmidt condensations, and a Biginelli reaction. In each case it is possible to monitor the reactions and also in one case, by means of a calibration curve, determine product conversion from Raman spectral data as corroborated by data obtained using NMR spectroscopy.

## Introduction

Continuous-flow processing is used in the chemical industry on production scales. In a research and development setting, there has been increasing interest in using flow chemistry on smaller scales. To this end, a wide range of companies now produce equipment for both micro- and mesofluidic flow chemistry [1–2]. Some of the advantages of these devices are increased experimental safety, easy scale-up and thorough mixing of reagents [3–7]. It is not surprising, therefore, that a wide range of synthetic chemistry transformations have been reported using this equipment [8–9]. When it comes to evaluating the outcome of reactions performed using flow chemistry and optimizing reaction conditions, one option is to use inline product analysis. This opens the avenue for fast, reliable assay in comparison with the traditional approach in which performance is evaluated based on offline product analysis. When interfaced with microreactors, inline analysis has taken significant strides in recent years [7,10]. Spectroscopic tools such as infrared [11–15], UV–visible [16–18], NMR [19–20], Raman [21–25], and mass spectrometry [26–27] have all been interfaced with success. There have been less reports when it comes to mesoflow systems. Perhaps most developed is the area of infrared monitoring. The now ubiquitous ReactIR equipment has been interfaced with commercially available flow equipment to allow for real-time analysis of reactions and on-the-fly optimization of conditions [28–30].

In our laboratory we have had success interfacing a Raman spectrometer with a scientific microwave unit [31]. This has allowed us to monitor reactions from both a qualitative [32–35] and quantitative [36–37] perspective. A recent report of the use of Raman spectroscopy for monitoring a continuous-flow palladium-catalyzed cross-coupling reaction [38] sparked our interest in interfacing our Raman spectrometer with one of our continuous-flow units and employing it for inline reaction monitoring of a number of key medicinally-relevant organic transformations. Our results are presented here.

## Results and Discussion

### Interfacing the spectrometer to the flow unit

**Figure 1:**
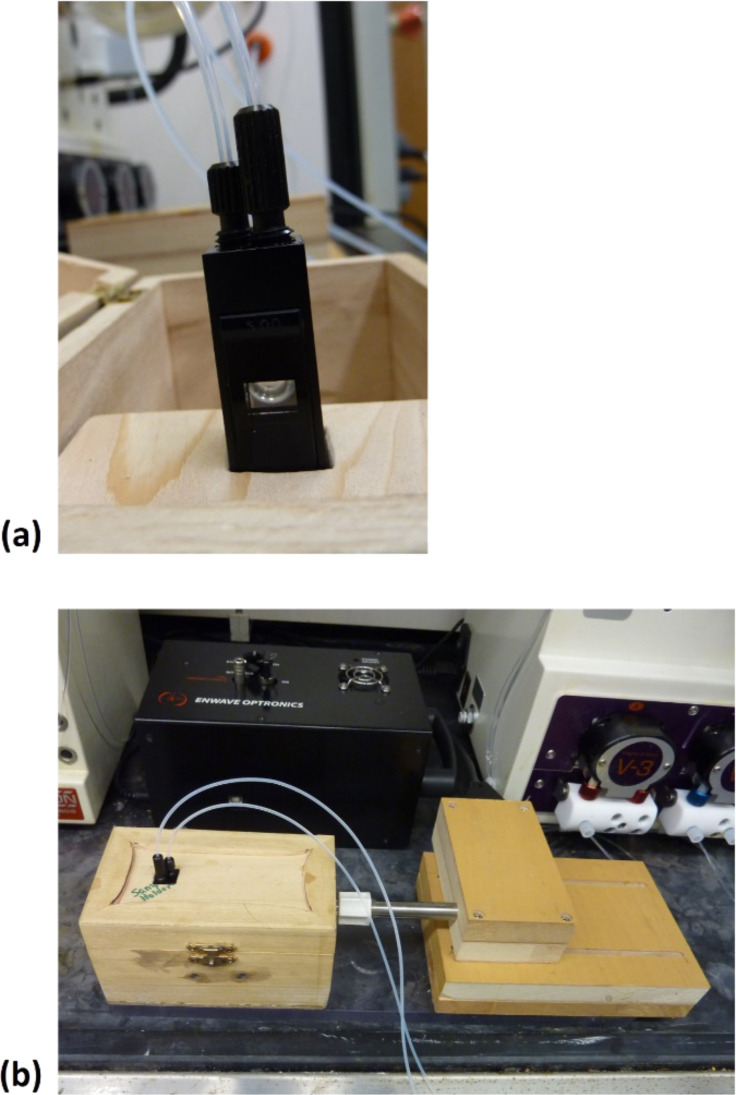
(a) Flow cell and (b) Raman interface used in the present study.

In interfacing our Raman spectrometer with a continuous-flow reactor, our objective was to use a similar approach to that which proved successful when using microwave heating. Borosilicate glass is essentially “Raman transparent”. Therefore reactions could be monitored by placing a Raman probe near the reaction vessel, without requirement to place any parts of the spectrometer inside the reaction vessel. The exposure of metallic components to the microwave field was avoided using a quartz light-pipe extending both the excitation laser and the acquisition fiber optic components of the spectrometer almost without any loss of light. The optimum distance of the light-pipe to the outside wall of the reaction vessel was found to be approximately 0.5 mm. Moving to our continuous-flow reactor, we decided to place the spectroscopic interface just after the back-pressure regulator assembly. This meant that we did not need to engineer a flow cell capable of holding significant pressure. Instead we used an off-the-shelf flow cell traditionally used in conjunction with other spectroscopic monitoring tools. The cell had screw-threaded inlet and outlet tubes of the same diameter as the tubing of the flow unit (i.d. 1 mm). The sample chamber had a width of 6.5 mm, height of 20 mm and a path length of 5 mm giving the cell a nominal internal volume of 0.210 mL (Figure 1a). We built an assembly to allow us to hold the cell in a fixed location and vary the distance of the quartz light-pipe so as to optimize the Raman signal intensity. The apparatus is shown in Figure 1b.

### Testing the interface: The synthesis of 3-acetylcoumarin

**Scheme 1:**
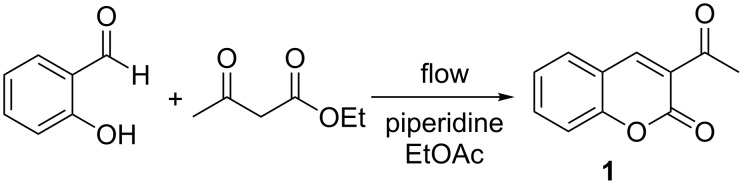
The reaction between salicylaldehyde and ethyl acetoacetate to form 3-acetyl coumarin (**1**).

As our first reaction for study, we selected the piperidine-catalyzed synthesis of 3-acetylcoumarin (**1**) from salicylaldehyde with ethyl acetoacetate (Scheme 1). We had extensive experience of monitoring this reaction both qualitatively [32] and quantitatively [36] when using microwave heating so believed it would be a good starting point for our present study. The reaction works well when using ethyl acetate as the solvent. However, **1** is not completely soluble at room temperature. To overcome potential clogging of the back-pressure regulator as well as mitigating the risk of having solid particles in the flow cell (which would perturb signal acquisition), we leveraged a technique we developed for this and other reactions previously [39]. Once the reaction stream has exited the heated zone, it is intercepted with a flow of a suitable organic solvent. This solubilizes the product and allows it to pass through the back-pressure regulator unimpeded. In the case of **1**, we intercept the product stream with a flow of acetone.

**Figure 2:**
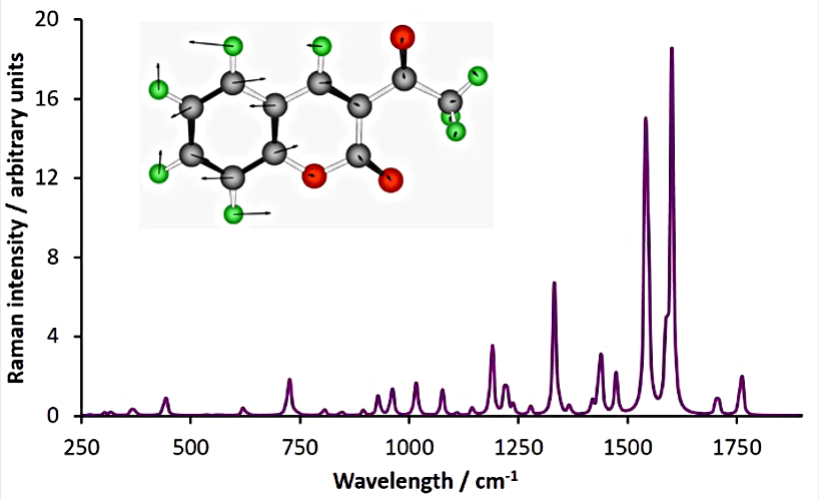
The Raman spectrum of 3-acetylcoumarin (**1**) generated using Gaussian 09 [40] at the B3LYP/6-31g(d) level of theory. The inset molecule illustrates the stretching mode responsible for the signal calculated at 1602 cm^−1^ (actual: 1608 cm^−1^).

**Figure 3:**
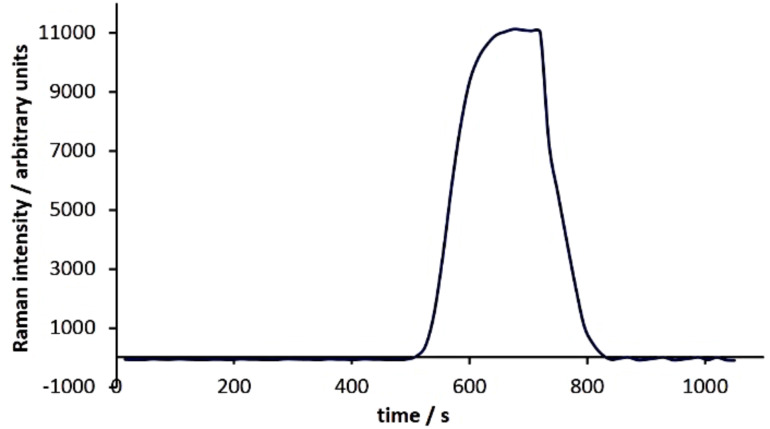
Monitoring an aliquot of 3-acetyl coumarin (**1**) as it passes through the flow cell (scan time = 15 s, integration = 10 s).

Our first objective was to determine whether we could observe spectroscopically a slug of the coumarin passing through the flow cell. The Raman spectrum of **1** (Figure 2) exhibits strong Raman-active stretching modes at 1608 cm^−1^ and 1563 cm^−1^ while the salicylaldehyde and ethyl acetoacetate starting materials exhibit minimal Raman activity in this area. As a result, we chose to monitor the 1608 cm^−1^ signal. To mimic a product mixture, we pumped a solution of **1** in acetone through our flow reactor, intercepted it with an equal volume of ethyl acetate and passed this mixture through the flow cell. We recorded a Raman spectrum every 15 s in an automated fashion as the coumarin passed through the cell by using the “continuous-scan” function on our spectrometer. By subtracting the spectrum of the solvent mixture (1:1 ethyl acetate:acetone) from the spectra recorded, we were able to clearly see the growth of the signal due to **1** followed by a plateau as it passed through the cell and then a drop back to the baseline as the final aliquot exited (Figure 3).

**Figure 4:**
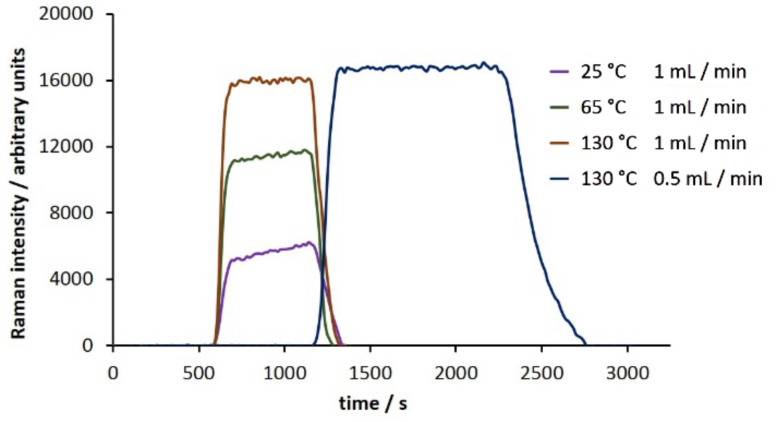
Monitoring the conversion of salicylaldehyde and ethyl acetoacetate to 3-acetylcoumarin (**1**) across a range of reaction conditions (scan time = 15 s, integration = 10 s).

Knowing we could observe the product as it passed through the flow cell, we next performed the complete reaction. As a starting point, we chose as conditions a flow rate of 1 mL/min through a 10 mL PFA coil at room temperature. We were indeed able to monitor the reaction as shown in Figure 4. In an effort to optimize the reaction conditions, we varied both the temperature of the reactor coil and also the flow rate, monitoring each run and then compiling the data (Figure 4). While increasing the reaction temperature to 130 °C led to a marked increase in product conversion, reducing the flow rate from 1 mL/min to 0.5 mL/min at this temperature did not have a significant impact on the outcome of the reaction.

**Figure 5:**
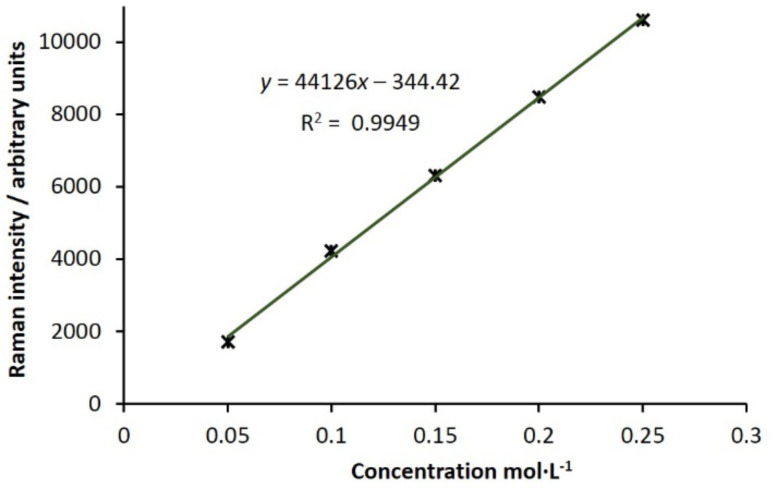
Plot of Raman intensity of the peak arising at 1608 cm^-1^ vs concentration of 3-acetyl coumarin (**1**), yielding a straight line, y = mx + b; m = Raman intensity·M^−1^ of **1**.

In an attempt to quantify product conversion, we needed next to obtain a calibration curve to allow us to convert units of Raman intensity to units of concentration in standard terms. To achieve this, we passed solutions of various concentrations of 3-acetylcoumarin (**3**) in ethyl acetate/acetone through the flow cell and collected the Raman spectrum. When the signal intensity at 1608 cm^−1^ is plotted against concentration, after subtraction of signals due to the solvent, the result is a straight line (Figure 5).

The Stokes shift (which is being monitored) is inversely proportional to the temperature. Since the flow cell is situated after the product mixture exits the heated zone and because of the very efficient heat transfer observed using narrow-gauge tubing, the product mixture was essentially at room temperature by the time it passed through the flow cell. As a result, it was not deemed necessary to involve a scaling factor to account for temperature effects.

With the appropriate calibration curve in hand, we were able to obtain product conversion values for each set of reaction conditions screened, taking into account the fact that the product concentration is halved by the interception with acetone. To determine their accuracy, we also determined product conversion using NMR spectroscopy. Comparison of the values shows a good correlation (Table 1).

**Table 1:** Comparison of product conversion values obtained from Raman spectra with those obtained using NMR spectroscopy for the conversion of salicylaldehyde and ethyl acetoacetate to 3-acetylcoumarin (**1**).

Conditions	Raman monitoring			NMR
	
	Concentration of **1** when diluted with acetone (mol L^–1^)	Concentration of **1** after normalizing for dilution by acetone (mol L^−1^)	Conv. (%)	Conv. (%)

25 °C, 1 mL/min	0.125	0.25	25	22
65 °C, 1 mL/min	0.27	0.55	55	58
130 °C, 1 mL/min	0.37	0.74	74	79
130 °C, 0.5 mL/min	0.39	0.78	78	80

### Expanding the technique to other reactions

#### The Knovenagel condensation

**Scheme 2:**
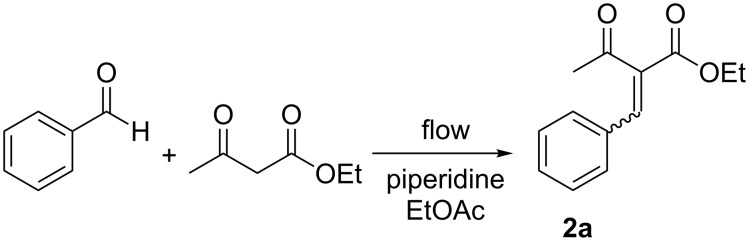
The Knoevenagel condensation of benzaldehyde and ethyl acetoacetate to yield (*Z*)-ethyl 2-benzylidene-3-oxobutanoate (**2a**).

We turned our attention next to the Knoevenagel condensation of ethyl acetoacetate with a range of aromatic aldehydes (Scheme 2). Our objective was to optimize conditions using one aldehyde substrate spectroscopically from a qualitative standpoint and then screen other examples. We chose benzaldehyde as our initial substrate, ethyl acetate as the solvent and piperidine as a base catalyst. In order to determine the optimal spectral frequency at which to monitor we wanted to find a quick way to derive the Raman spectrum of the product **2a**. As was the case with **1**, this could be achieved computationally using Gaussian 09 at the B3LYP/6-31g(d) level of theory [40], and a signal at 1598 cm^−1^ selected for monitoring.

**Figure 6:**
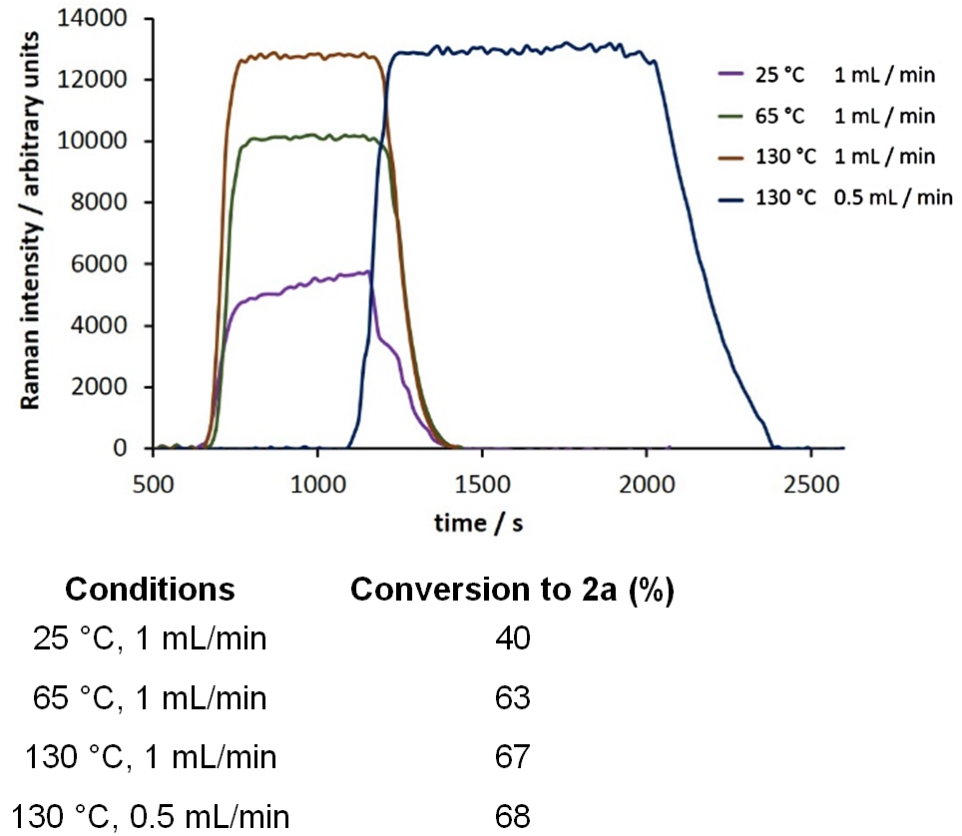
Monitoring the conversion of benzaldehyde and ethyl acetoacetate to (*Z*)-ethyl 2-benzylidene-3-oxobutanoate (**2a**) across a range of reaction conditions (scan time = 15 s, integration = 10 s).

Performing the reaction across a range of conditions, flowing the reaction mixture at 1 mL/min through the 10 mL coil heated to 130 °C proved to be optimal (Figure 6). A 67% conversion to **2a** was obtained, as determined by GC analysis. Purification of the product mixture gave a 60% isolated yield of the *Z*-isomer of **2a**. Using these optimized reaction conditions, we screened three para-substituted aldehyde substrates (Table 2). As expected, placing an electron-donating methoxy group on the aromatic ring led to lower product conversion as compared to benzaldehyde (Table 2, entry 2). A methyl- or fluoro-substituent has little effect on the outcome of the reaction (Table 2, entries 3 and 4).

**Table 2:** Product conversion obtained for four aldehyde substrates in the Knoevenagel reaction with ethyl acetoacetate.

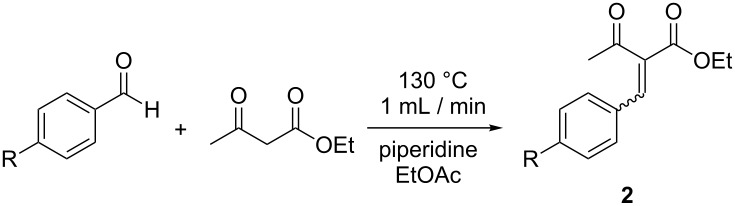		

R	Product	Conv. (%)

H	**2a**	67 (60)^a^
OMe	**2b**	53
Me	**2c**	66
F	**2d**	63

^a^Isolated yield.

#### The Claisen–Schmidt condensation

**Scheme 3:**
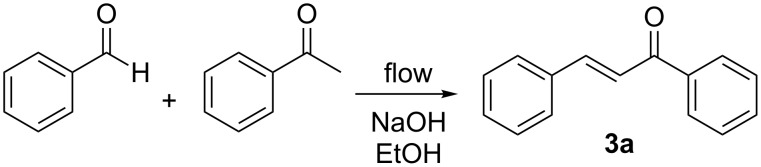
Claisen-Schmidt condensation of benzaldehyde with acetophenone to yield chalcone, **3a**.

**Figure 7:**
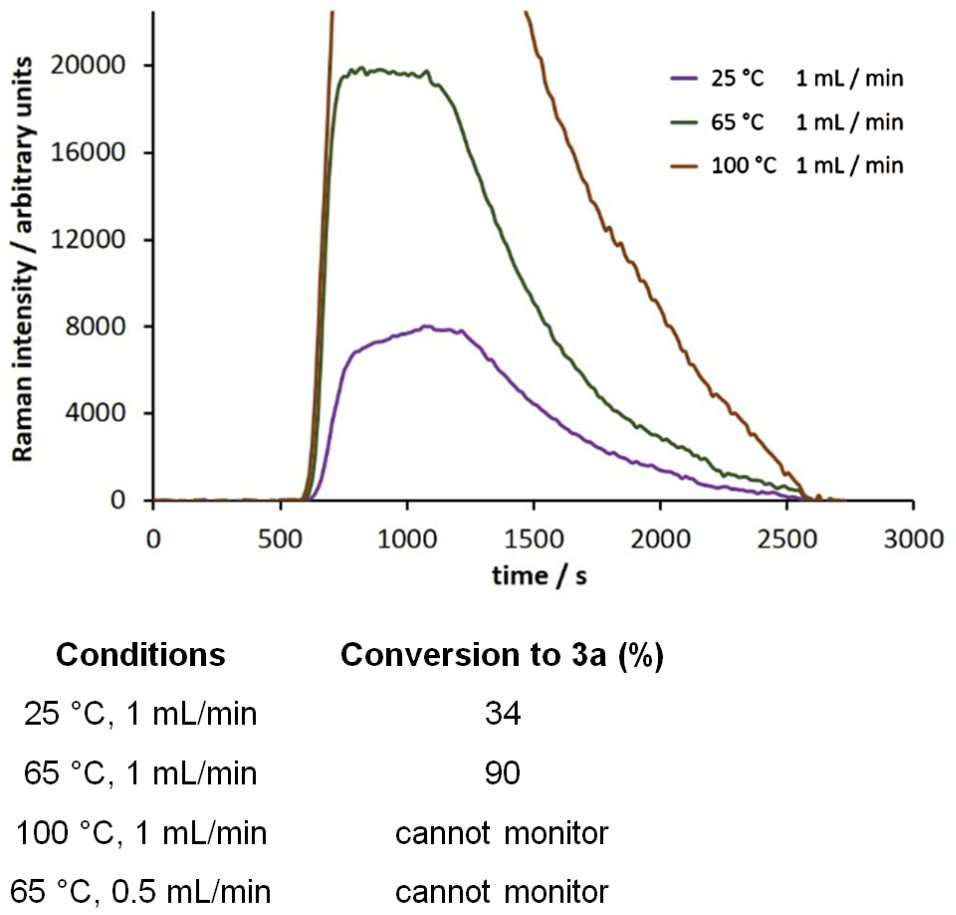
Monitoring the conversion of benzaldehyde with acetophenone to chalcone, **3a**, across a range of reaction conditions (scan time = 15 s, integration = 10 s).

We moved next to study the Claisen–Schmidt condensation of benzaldehyde with acetophenone to yield chalcone (Scheme 3). Chalcones display interesting biological properties such as antioxidant, cytotoxic, anticancer, antimicrobial, antiprotozoal, antiulcer, antihistaminic, and anti-inflammatory activity [41]. They are also intermediates on the way to highly fluorescent cyanopyridine and deazalumazine dyes [42]. The calculated Raman spectrum of the product **3a** shows a very strong signal at 1604 cm^−1^ which was selected for monitoring. Using sodium hydroxide as the catalyst, the reaction was monitored under a range of reaction conditions (Figure 7). We fast discovered that at temperatures in excess of 65 °C we observed decomposition or else formation of a highly fluorescent byproduct, as evidenced by collapse of the Raman spectrum. We also observed a significant “tail” on the plot of signal intensity at 1604 cm^−1^ vs time. We attribute this to the fact that the chalcone product is very highly Raman active and even a trace in the flow cell can be readily detected. It does however highlight the fact that there may be both significant dispersion along the length of the reactor and the product is slow in clearing the flow cell. Dispersion is the consequence of laminar flow and some of the material takes longer to travel through the reactor than the rest. Thus, when a flow reactor is used to process a finite volume of reagents, the leading and trailing ends of the product emerging from the end of the reactor will have mixed to some extent with the solvent that preceded or followed it. This means that there are zones at the leading and trailing ends of the product stream in which the concentration of product is variable. Our optimal conditions for the reaction were heating at 65 °C with a flow rate of 1 mL/min, this corresponding to a product conversion of 90%, as determined by GC analysis. Performing the reaction under these conditions using three substituted benzaldehydes as substrates, we obtained product conversions of 66−98% depending on how electron rich or deficient the aromatic ring of the aldehyde was (Table 3).

**Table 3:** Product conversion obtained for four aldehyde substrates in the Claisen-Schmidt reaction with acetophenone.

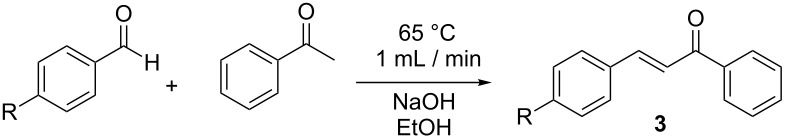		

R	Product	Conv. (%)

H	**3a**	90
OMe	**3b**	66
Me	**3c**	84
F	**3d**	98 (90)^a^

^a^Isolated yield.

#### The Biginelli reaction

**Scheme 4:**
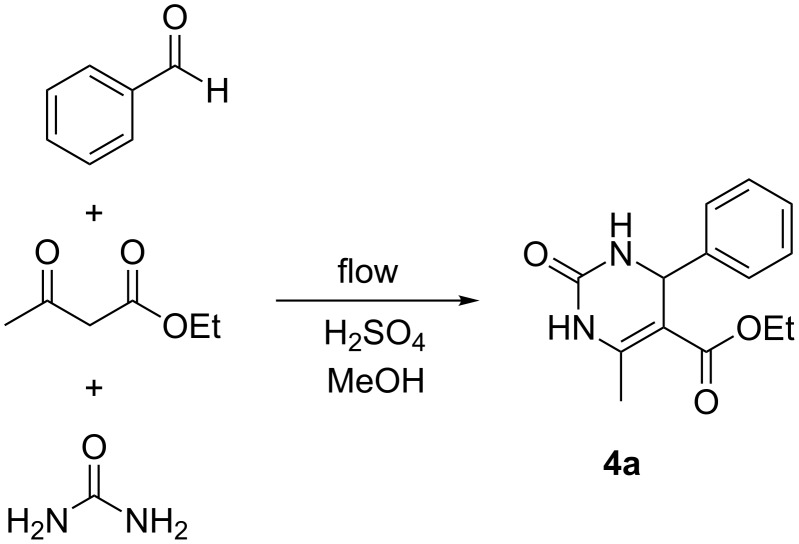
The Biginelli cyclocondensation of benzaldehyde, ethyl acetoacetate, and urea to yield 5-ethoxycarbonyl-6-methyl-4-phenyl-3,4-dihydropyrimidin-2(1*H*)-one (**4a**).

**Figure 8:**
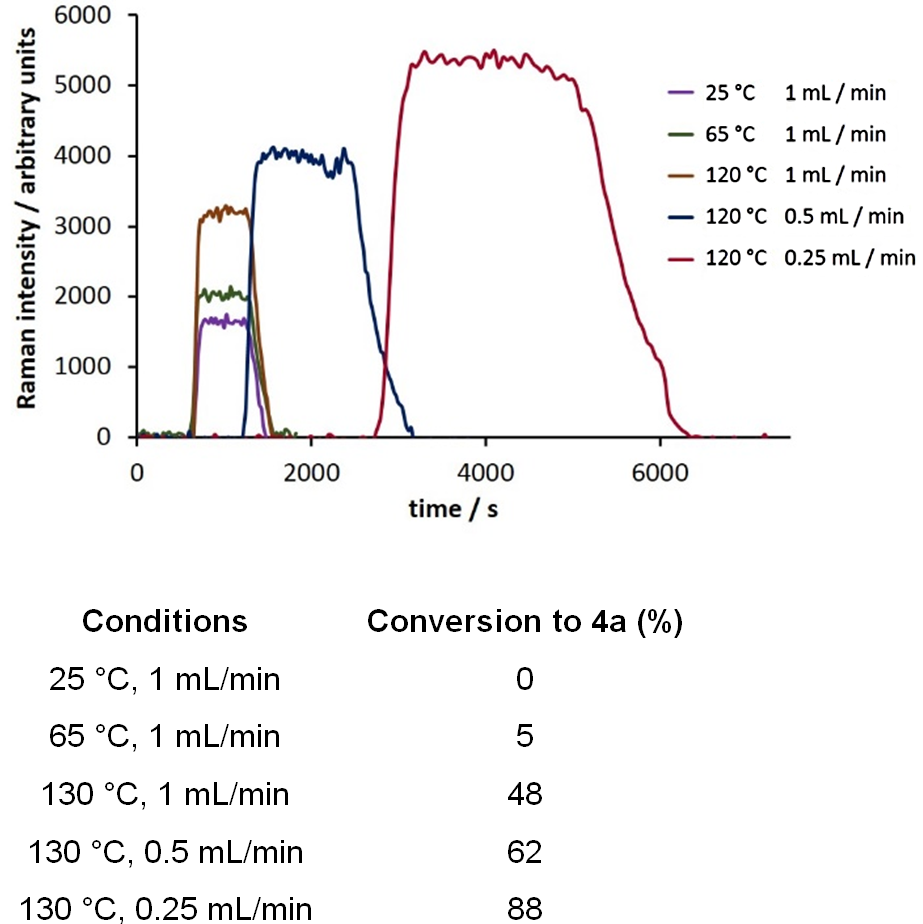
Monitoring the conversion of benzaldehyde, ethyl acetoacetate, and urea to 5-ethoxycarbonyl-6-methyl-4-phenyl-3,4-dihydropyrimidin-2(1*H*)-one (**4a**) across a range of reaction conditions (scan time = 15 s, integration = 10 s).

As our final reaction for study, we turned to the Biginelli reaction (Scheme 4) [43–48]. This acid-catalyzed cyclocondensation of urea, β-ketoesters and aromatic aldehydes to yield dihydropyrimidines has received significant attention, these products having pharmacological activity including calcium channel modulation, mitotic kinesin Eg5 inhibition, and antiviral and antibacterial activity [49–50]. The Biginelli reaction has been performed in flow previously as a route to densely functionalized heterocycles using HBr generated in a prior step as the catalyst for the reaction [51]. Copper catalysis has also been used in flow mode for preparing PEG-immobilized dihydropyrimidines [52]. We decided to screen a set of conditions for the reaction of benzaldehyde, ethyl acetoacetate and urea catalyzed by sulfuric acid (Figure 8).

The calculated Raman spectrum of the product, **4a**, shows a strong signal at 1598 cm^−1^ which was selected for monitoring. Using a catalyst loading of 10 mol % and a flow rate of 1 mL/min, we monitored the reaction over a temperature range from 25–120 °C. Seeing that the reaction did not reach completion within the 10 min in the heated zone, we then repeated the process at lower flow rates; first to 0.5 mL/min and then 0.25 mL/min. Our optimal conditions as determined by Raman monitoring were heating at 120 °C with a flow rate of 0.25 mL/min, this corresponding to a product conversion of 89%, as determined by GC analysis, and a product yield of 78% after purification. Performing the reaction using three other aldehyde substrates resulted in similar product conversions (Table 4).

**Table 4:** Product conversion obtained for four aldehyde substrates in the Biginelli reaction with ethyl acetoacetate and urea.

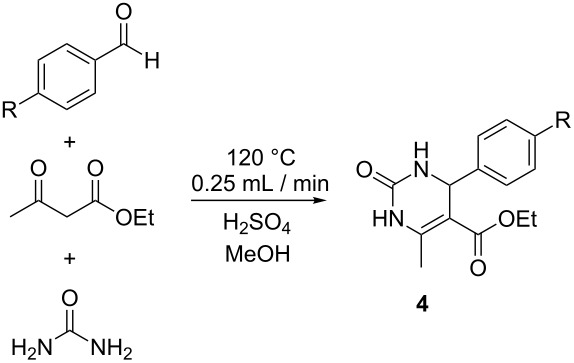		

R	Product	Conv. (%)

H	**4a**	88 (78)^a^
OMe	**4b**	85
Me	**4c**	87
F	**4d**	91

^a^Isolated yield.

## Conclusion

In conclusion, we describe here an apparatus for real-time Raman monitoring of reactions performed using continuous-flow processing. We assess its capability by studying four reactions. We find that it is possible to monitor reactions and also, by means of a calibration curve, determine product conversion from Raman spectral data as corroborated by data obtained using NMR spectroscopy. Work is now underway to expand the scope of the method to other classes of useful reactions.

## Experimental

### General experimental

All reagents are used as received from the various vendors without purification. Sodium sulfate, MeOH, EtOH, EtOAc, DMF and Et_2_O (ACS Grade and reagent grade), were purchased from Sigma-Aldrich and used without further purification. Deuterated NMR solvents (CDCl_3_) were purchased from Cambridge Isotope Laboratories. CDCl_3_ stored over 4Å molecular sieves and K_2_CO_3_. NMR Spectra (^1^H, ^13^C, ^19^F) were performed at 298 K on either a Bruker DRX-400 MHz NMR, or Bruker Avance 500 MHz NMR. ^1^H NMR Spectra obtained in CDCl_3_ were referenced to residual non-deuterated chloroform (7.26 ppm) in the deuterated solvent. ^13^C NMR Spectra obtained in CDCl_3_ were referenced to chloroform (77.3 ppm). ^19^F NMR spectra were referenced to hexafluorobenzene (−164.9 ppm) [53]. Reactions were monitored by an Agilent Technologies 7820A Gas Chromatograph attached to a 5975 Mass Spectrometer or ^1^H NMR. Flash chromatography and silica plugs utilized Dynamic Adsorbants Inc. Flash Silica Gel (60Å porosity, 32-63 µm).

#### Apparatus configuration

The Raman system used was an Enwave Optronics Spectrometer, Model EZRaman-L [32]. The continuous-flow unit used was a Vapourtec E-series. A Starna 583.65.65-Q-5/Z20 flow-cell (width: 6.5 mm, height: 20 mm, path length: 5 mm) was placed inline after the back-pressure regulator using 1 mm i.d. PFA tubing (the void volume between the flow reactor and the flow cell was 4.79 mL). The flow cell was secured in place in a custom-made box and the fiber-optic probe from the spectrometer inserted so it touched the wall of the flow cell. During a reaction, spectral data was recorded at pre-determined time intervals using the EZ Raman software provided with the instrument. The data was then exported to Excel for processing.

#### Typical procedure for monitoring the formation of 3-acetylcoumarin (1)

**Performing the reaction:** Into a 50 mL volumetric flask was added salicylaldehyde (6.106 g, 50 mmol, 1 equiv) and ethyl acetoacetate (6.507 g, 50 mmol, 1 equiv). Ethyl acetate was added to bring the total volume to 50 mL (1 M) and the reagents were thoroughly mixed. An aliquot of this solution (10 mL) was transferred to a 20 mL vial equipped with a Teflon-coated stir bar. The flow reactor was readied using the equipment manufacturer’s suggested start-up sequence. Ethyl acetate was pumped at 1 mL/min to fill the reactor coil. The back-pressure regulator was adjusted to 7 bar and the reactor coil heated to 65 °C. After the heating coil, the product stream was intercepted with a stream of acetone (1 mL/min) by means of a T-piece to ensure complete solubility of the product. The Raman probe was inserted into the box containing the flow cell and was properly focused. A background scan of the ethyl acetate/acetone solvent system was taken. This background was then automatically subtracted from all subsequent scans, thereby removing any signals from the solvent. The Raman spectrometer was set to acquire a spectrum every 15 s throughout the run, with 10 s integration time, boxcar = 3, and average = 1. When the flow unit was ready, piperidine (0.099 mL, 0.1 mmol, 0.1 equiv.) was injected all at once into the vial containing the reagents and, after mixing for 15 s, the reaction mixture was loaded into the reactor at a flow rate of 1 mL/min. After the reaction mixture had been completely loaded into the reactor, ethyl acetate was again pumped through the coil at 1 mL/min. After the product had been fully discharged from the flow cell, the scans were halted. While the product mixture was passing through the flow cell, a drop of the exit stream was removed and an NMR spectrum recorded to obtain product conversion for comparison with data obtained by Raman spectroscopy. NMR conversions were determined by comparing signals from the starting salicylaldehyde (9.84 ppm) and the coumarin product (8.45 ppm) [36].

**Obtaining a relationship between signal strength and concentration:** To obtain a calibration curve, spectra of 3-acetylcoumarin in 1:1 ethyl acetate/acetone were recorded at a range of concentrations by passing the solutions through the flow cell. A plot of signal strength due to the peak at 1608 cm^−1^ versus concentration of **1** was constructed (Figure 5). From this, units of Raman intensity could be converted to units of concentration in standard terms and hence product conversion determined.

#### Typical procedure for monitoring the Knoevenagel reaction

An analogous approach was used to prepare the Knoevenagel product as for the case of **1**, benzaldehyde (5.306 g, 50 mmol, 1 equiv) being used in place of salicylaldehyde and there being no need for acetone interception of the product mixture. The Raman spectrometer was programmed to take continuous scans using the same parameters as in the case of **1**. After the product had been fully discharged from the flow cell, the scans were halted. The resulting clear yellow solution was poured over aqueous 2 M HCl and extracted with ethyl acetate. The combined organic layers were washed with brine, dried over sodium sulfate, and the solvent was removed in vacuo by rotary evaporation affording the crude product. The crude product was loaded on a 15-cm silica gel column (55 g silica gel) and a gradient eluting system (99:1, 95:5, 90:10; Hex:EtOAc) was used to obtain (*Z*)-ethyl 2-benzylidene-3-oxobutanoate (**2a**, 1.3095 g, 60%) as a clear yellow oil. ^1^H NMR (CDCl_3_, 400 MHz) δ ppm 1.26 (t, *J* = 7.21 Hz, 3H), 2.41 (s, 3H), 4.32 (q, *J* = 7.09 Hz, 2H), 7.33–7.41 (m, 3H), 7.42–7.47 (m, 2H), 7.56 (s, 1H); ^13^C NMR (CDCl_3_, 100 MHz) δ ppm 14.08 (CH_3_), 26.74 (CH_3_), 61.92 (CH_2_), 129.06 (CH), 129.74 (CH), 130.93 (CH), 133.17 (C), 134.88 (C), 141.48 (CH), 168.00 (C), 194.87 (C) [54].

#### Typical procedure for monitoring the Claisen–Schmidt reaction

Into a 50 mL volumetric flask was added 4-fluorobenzaldehyde (1.551 g, 12.5 mmol, 1 equiv) and acetophenone (1.637 g, 12.5 mmol, 1 equiv). Ethanol was added to bring the total volume to 50 mL (0.25 M) and the reagents were thoroughly mixed. An aliquot of this solution (10 mL) was transferred to a 20 mL vial equipped with a Teflon-coated stir bar. The flow reactor was readied using the equipment manufacturer’s suggested start-up sequence. Ethanol was pumped at 0.5 mL/min to fill the reactor coil. The back-pressure regulator was adjusted to 7 bar and the reactor coil heated to 65 °C. After the heating coil, the product stream was intercepted with a stream of acetone (0.5 mL/min) by means of a T-piece to ensure complete solubility of the product. The Raman spectrometer was configured as in the case of monitoring formation of **1**. When the flow unit was ready, 2 M NaOH (0.125 mL, 0.25 mmol) was injected all at once and after mixing for 15 s the reaction mixture was loaded into the reactor coil at a flow rate of 0.5 mL/min. After the reaction mixture had been completely loaded into the reactor, ethanol was again pumped through the coil at 0.5 mL/min. After the product had been fully discharged from the flow cell, the scans were halted. The yellow product solution was poured into a beaker containing ice (100 g) causing an immediate precipitation of the product. To ensure complete precipitation, the solution was stirred at 0 °C. The solid product was collected via vacuum filtration and washed with cold ethanol. The material was dried in air to yield (*E*)-3-(4-fluorophenyl)-1-phenylprop-2-en-1-one, (**3d**, 0.5421 g, 91%) as a pale yellow solid. ^1^H NMR (CDCl_3_, 400 MHz) δ ppm 7.11 (t, *J* = 8.68 Hz, 2H), 7.46 (d, *J* = 15.89 Hz, 1H), 7.49–7.55 (m, 2H), 7.56–7.69 (m, 3H), 7.78 (d, *J* = 15.65 Hz, 1H), 8.02 (d, *J* = 7.34 Hz, 2H); ^13^C NMR (CDCl_3_, 100 MHz) δ ppm 116.42 (d, *J*_C-C-F_ = 22.01 Hz, CH), 122.09 (d, *J*_C-C-C-C-F_ = 2.20 Hz, C), 128.76 (s, 10C), 128.94 (s, 9C), 130.62 (d, *J*_C-C-C-F_ = 8.80 Hz, CH), 131.45 (d, *J*_C-C-C-C-C-F_ = 3.67 Hz, CH), 133.12 (C), 138.43 (C), 143.78 (CH), 164.35 (d, *J*_C-F_ = 250.89 Hz, C), 190.59 (C); ^19^F NMR (CDCl_3_, 377 MHz) δ ppm −113.59, −111.32 (m) [55–56].

#### Typical procedure for monitoring the Biginelli reaction

In a 50 mL volumetric flask was dissolved urea (3.003 g, 50 mmol, 1 equiv.) in methanol (~30 mL). Into the flask was then added benzaldehyde (1.306 g, 50 mmol, 1 equiv) and ethyl acetoacetate (6.507 g, 50 mmol, 1 equiv). Methanol was added to bring the total volume to 50 (1 M) and the reagents were thoroughly mixed. An aliquot of this solution (10 mL) was transferred to a 20 mL vial equipped with a Teflon-coated stir bar. The flow reactor was readied using the equipment manufacturer’s suggested start-up sequence. Methanol was pumped at 0.25 mL/min to fill the reactor coil. The back-pressure regulator was adjusted to 7 bar and the reactor coil heated to 120 °C. After the heating coil, the product stream was intercepted with a stream of *N*,*N*-dimethylformamide (0.25 mL/min) by means of a T-piece to ensure complete solubility of the product. The Raman spectrometer was set to acquire a spectrum every 25 s, with 20 s integration time, boxcar = 3, and average = 1. When the flow unit was ready, 6 M H_2_SO_4_ (0.2 mL, 0.1 equiv) was injected all at once and after mixing for 15 s the reaction mixture was loaded into the reactor coil at a flow rate of 0.25 mL/min. After the reaction mixture had been completely loaded into the reactor, methanol was again pumped through the coil at 0.25 mL/min. After the product had been fully discharged from the flow cell, the scans were halted. The reaction mixture was transferred to a separatory funnel, diluted with diethyl ether and quenched with satd. sodium bicarbonate (100 mL) and deionized water (100 mL). The layers were separated and the aqueous layer was extracted with diethyl ether (3 × 100 mL). The combined organic layers were washed with brine (2 × 100 mL) and dried over sodium sulfate. The solvent was removed in vacuo by rotary evaporation affording the crude product. The resulting solid was transferred to a filter funnel and was washed with cold methanol. The solid was isolated and air dried to afford 5-ethoxycarbonyl-6-methyl-4-phenyl-3,4-dihydropyrimidin-2(1*H*)-one, (**4a**, 2.030 g, 78%) as a fluffy white solid. ^1^H NMR (DMSO-*d*_6_, 500 MHz) δ ppm 1.18 (s., 3H), 2.34 (s., 3H), 3.67–4.60 (m, 2H), 5.24 (s., 1H), 7.34 (s., 5H), 7.80 (s., 1H), 7.74 (s, 1H), 9.26 (s, 1H); ^13^C NMR (DMSO-*d*_6_, 125 MHz) δ ppm 14.5 (CH_3_), 18.2 (CH_3_), 54.4 (CH), 59.6 (CH_2_), 99.7 (C), 126.7 (CH), 127.7 (CH), 128.8 (CH), 145.3 (C), 148.8 (C), 152.6 (C), 165.8 (C) [57].

## Supporting Information

File 1 NMR spectra of the isolated products (**1**, **2a**, **3d**, **4a**), further experimental information, and pictures of the Raman interface and Cartesian coordinates of the stationary points.
