# Supplementary material for: Raman spectroscopy as a tool for monitoring mesoscale continuous-flow organic synthesis: Equipment interface and assessment in four medicinally-relevant reactions
Source: Beilstein J Org Chem. 2013 Sep 11;9:1843–52. doi: 10.3762/bjoc.9.215 (PMC3778413; doi:10.3762/bjoc.9.215)

Supporting Information

for

Raman spectroscopy as a tool for monitoring mesoscale continuous-flow organic synthesis: Equipment interface and assessment in four medicinally-relevant reactions

Trevor A. Hamlin and Nicholas E. Leadbeater*

Department of Chemistry, University of Connecticut, 55 North Eagleville Road, Storrs, CT 06269, USA

Email: Nicholas E. Leadbeater - nicholas.leadbeater@uconn.edu

*corresponding author

NMR spectra of the isolated products (1, 2a, 3d, 4a), further experimental information, and pictures of the Raman interface and Cartesian coordinates of the stationary points

Specifics of the Raman spectrometer / flow chemistry interface	S2
Photographs of the Raman spectrometer / flow chemistry interface	S3
Experimental details for the four reactions studied	S4–S11
Catesian Coordinates of All Stationary Points	S12–S15
^{1}H NMR spectra of isolated compounds (**1a**, **2a**, **3d**, **4a**)	S16–S19
^{13}C NMR spectra of isolated compounds (**1a**, **2a**, **3d**, **4a**)	S20–S23
^{19}F NMR spectra of **3d**	S24

Specifics of the Raman spectrometer / flow chemistry interface

The Raman system used was an Enwave Optronics Spectrometer, Model EZRaman-L (www.enwaveopt.com). The continuous-flow unit used was a Vapourtec E-series. A Starna 583.65.65-Q-5/Z20 flow-cell (width: 6.5 mm, height: 20 mm, path length: 5 mm) was placed in-line after the back-pressure regulator using 1mm i.d. PFA tubing. The flow cell was secured in place in a custom-made box and the fiber-optic probe from the spectrometer inserted so it touched the wall of the flow cell.

EXCITATION SOURCE: NIR, frequency stabilized, narrow linewidth diode laser at 785 nm. Laser power at sample ~200 mW. Linewidth < 2 cm^{-1}. Fiber-coupled laser output (100 µm, 0.22 NA).

FIBER-OPTIC PROBE: Permanently-aligned two single fiber combination 100 µm excitation fiber, 200 µm collection fiber (0.22 NA). Working distance: 8 mm (standard). Rayleigh rejection: O.D. > 7 at laser wavelength.

CCD DETECTOR: High sensitivity linear CCD array. Temperature regulated (at 13 °C) operation for long integration time and stable dark reference subtraction. Pixel Size: 14 µm x 200 µm (2048 pixels); 16 bit digitization.

SPECTROGRAPH: Symmetrical crossed Czerny-Turner design. Resolution: ~10 cm^{-1} at 785 nm. Excitation spectral coverage: 200 cm^{-1} to 2400 cm^{-1}. Built-in software calibration.

SYSTEM SOFTWARE: EZ Raman 3.5.4MAS. Data files exported into Microsoft Excel.

The flow system used in the study was a Vapourtec E-series (www.vapourtec.co.uk) equipped with a 10 mL PFA reactor coil.

The flow cell used for spectroscopic studies was a Starna 583.65.65-Q-5/Z20 cell (width: 6.5 mm, height: 20 mm, path length: 5 mm) (www.starnacells.com).

Photographs of the Raman spectrometer / flow chemistry interface

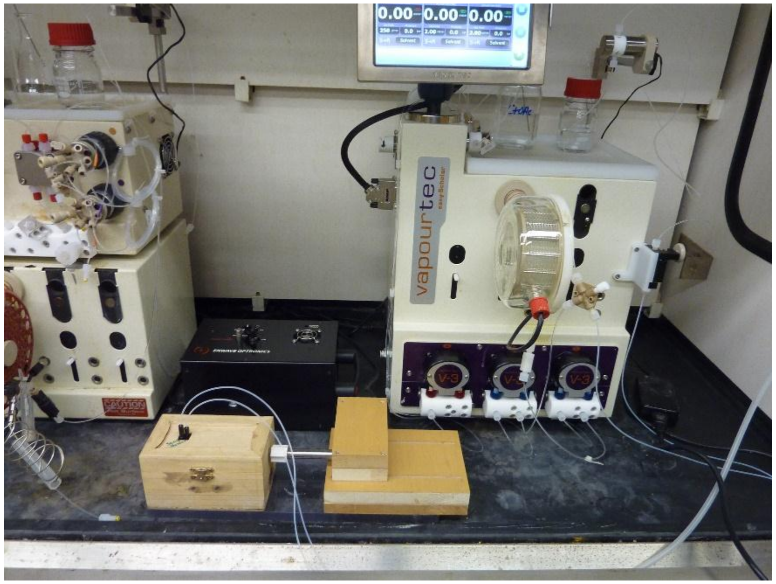

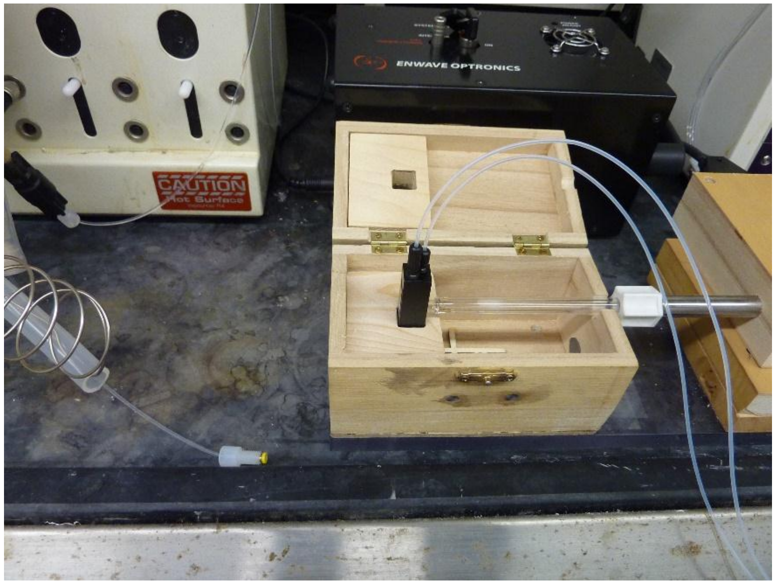

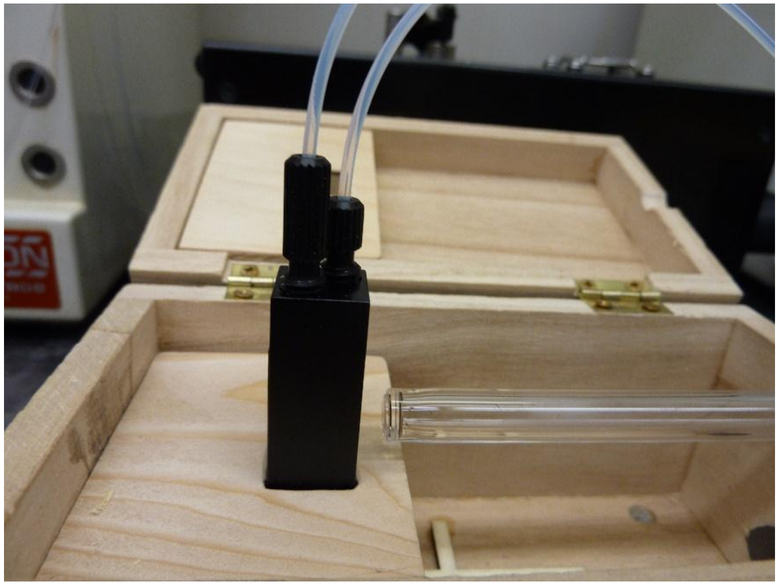

Experimental details for the four reactions studied

Preparation of coumarins

3-Acetylcoumarin (1a), compound 1 of the main article:

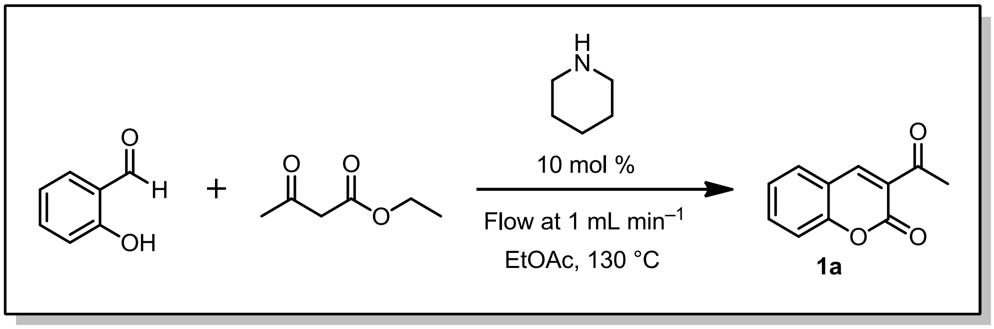

Into a 50 mL volumetric flask was added salicylaldehyde (6.106 g, 50 mmol, 1 equiv) and ethyl acetoacetate (6.507 g, 50 mmol, 1 equiv). Ethyl acetate was added to bring the total volume to 50 mL (1 M) and the reagents were thoroughly mixed. An aliquot of this solution (10 mL) was transferred to a 20 mL vial equipped with a Teflon-coated stir bar. The flow reactor was readied using the equipment manufacturer's suggested start-up sequence. Ethyl acetate was pumped at 1 mL/min to fill the reactor coil. The back-pressure regulator was adjusted to 7 bar and the reactor coil heated to 65 °C. After the heating coil, the product stream was intercepted with a stream of acetone (1 mL/min) by means of a T-piece to ensure complete solubility of the product. The Raman probe was inserted into the box containing the flow cell and was properly focused. A background scan of the ethyl acetate/acetone solvent system was taken. This background was then automatically subtracted from all subsequent scans, thereby removing any signals from the solvent. The Raman spectrometer was set to acquire a spectrum every 15 s throughout the run, with 10 s integration time, boxcar = 3, and average = 1. When the flow unit was ready, piperidine (0.099 mL, 0.1 mmol, 0.1 equiv) was injected all at once into the vial containing the reagents and, after mixing for 15 s, the reaction mixture was loaded into the reactor at a flow rate of 1 mL/min. After the reaction mixture had been completely loaded into the reactor, ethyl acetate was again pumped through the coil at 1 mL/ min. After the product had been fully discharged from the flow cell, the scans were halted. After the product had been fully discharged from the flow cell, the scans were halted and the resulting clear yellow solution was then concentrated in vacuo by rotary evaporation. The crude was transferred to a filter funnel and washed with cold hexanes. The product was recrystallized from ethanol to afford 3-acetylcoumarin **1a** (1.56 g, 85%) as a pale yellow solid.

^{1}H NMR ($CDCl_3$, 500 MHz) δ ppm 2.73 (s, 3 H), 7.31 - 7.40 (m, 2 H), 7.65 (ddd, *J* = 7.53, 4.37, 2.60 Hz, 2 H), 8.51 (s, 1 H).

^{13}C NMR ($CDCl_3$, 125 MHz) δ ppm 30.84 (CH_3), 117.00 (CH), 118.56 (C), 124.86 (CH), 125.27 (CH), 130.51 (CH), 134.68 (C), 147.74 (CH), 155.64 (C), 159.52 (C), 195.77 (C).

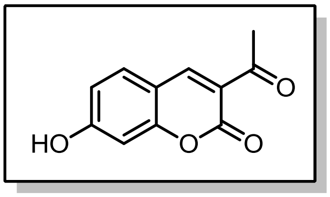

3-Acetyl-7-hydroxy-2*H*-chromen-2-one (1b)
(70% NMR conversion) was prepared according to the representative procedure for the synthesis of **1a** from 2,4-dihydroxybenzaldehyde (1.3812 g, 10 mmol).

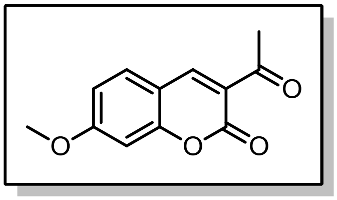

3-Acetyl-7-methoxy-2*H*-chromen-2-one (1c)
(73% NMR conversion) was prepared according to the representative procedure for the synthesis of **1a** from 2-hydroxy-4-methoxy benzaldehyde (1.5215 g, 10 mmol). One modification was made: intercept with acetone flowing at 3 mL/min.

Knoevenagel reaction

(*Z*)-Ethyl 2-benzylidene-3-oxobutanoate (2a):

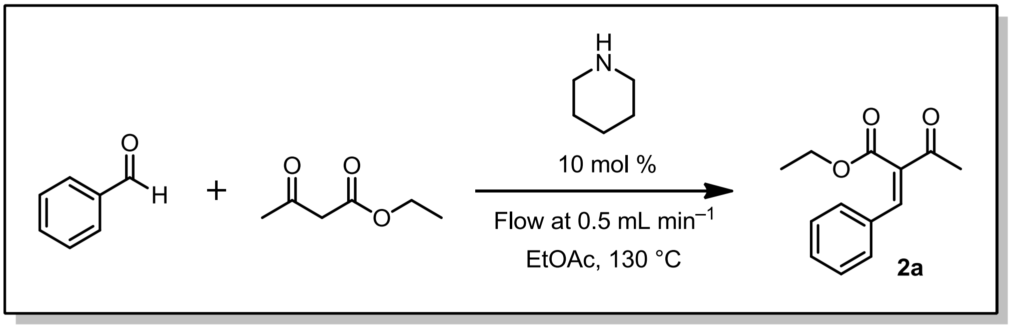

Into a 50 mL volumetric flask was added benzaldehyde (5.306 g, 50 mmol, 1 equiv) and ethyl acetoacetate (6.507 g, 50 mmol, 1 equiv). EtOAc was added to bring the total volume to 50 mL (1 M) and the reagents were thoroughly mixed. An aliquot of this solution (10 mL) was transferred to a 20 mL vial equipped with a Teflon-coated stir bar. The flow reactor was readied using the equipment manufacturer's suggested start-up sequence. Ethyl acetate was pumped at 1 mL/min to prime the system as the coil was heated to 130 °C. The back-pressure regulator was adjusted to 7 bar to prevent the solvent from boiling in the coil. The Raman probe was inserted into the box containing the flow cell and was properly focused. The background scan of the solvent system was taken that would be subtracted from all subsequent scans. Piperidine (0.099 mL, 0.1 mmol, 0.1 equiv) was injected all at once and, after mixing for 15 s, the reaction mixture was loaded into the reactor at a flow rate of 1 mL/min. Product collection was commenced immediately after this switch. After the reaction mixture had been completely loaded into the reactor, the reactor pump was set back to pumping ethyl acetate. The resulting clear yellow solution was poured over aqueous 2 M HCl and extracted with ethyl acetate. The combined organic layers washed with brine, dried over Na_2SO_4, and the solvent was removed in vacuo by rotary evaporation affording the crude product. The crude product was loaded on a 15-cm silica gel column (55 g silica gel) and a gradient eluting system (99:1, 95:5, 90:10; Hex:EtOAc) was used to obtain (*Z*)-ethyl 2-benzylidene-3-oxobutanoate, **2a** (1.3095 g, 60%) as a clear yellow oil.

^{1}H NMR ($CDCl_3$, 400 MHz) δ ppm 1.26 (t, *J* = 7.21 Hz, 3 H), 2.41 (s, 3 H), 4.32 (q, *J* = 7.09 Hz, 2 H), 7.33 - 7.41 (m, 3 H), 7.42 - 7.47 (m, 2 H), 7.56 (s, 1 H).

^{13}C NMR ($CDCl_3$, 100 MHz) δ ppm 14.08 (CH_3), 26.74 (CH_3), 61.92 (CH_2), 129.06 (CH), 129.74 (CH), 130.93 (CH), 133.17 (C), 134.88 (C), 141.48 (CH), 168.00 (C), 194.87 (C).

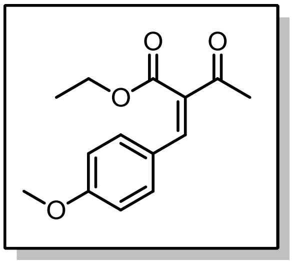

(*Z*)-Ethyl 2-(4-methoxybenzylidene)-3-oxobutanoate (2b)

(53% GC conversion) was prepared according to the representative procedure for the synthesis of **2a** from 4-methoxybenzaldehyde (1.3615 g, 10 mmol).

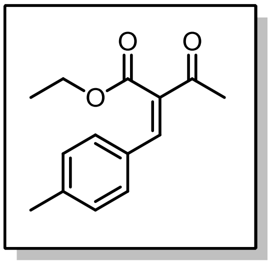

(*Z*)-Ethyl 2-(4-methylbenzylidene)-3-oxobutanoate (2c)
(66% GC conversion) was prepared according to the representative procedure for the synthesis of **2a** from 4-methylbenzaldehyde (1.2015 g, 10 mmol).

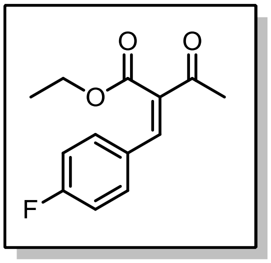

(*Z*)-Ethyl 2-(4-fluorobenzylidene)-3-oxobutanoate (2d)
(63% GC conversion) was prepared according to the representative procedure for the synthesis of **2a** from 4-fluorobenzaldehyde (1.2411 g, 10 mmol).

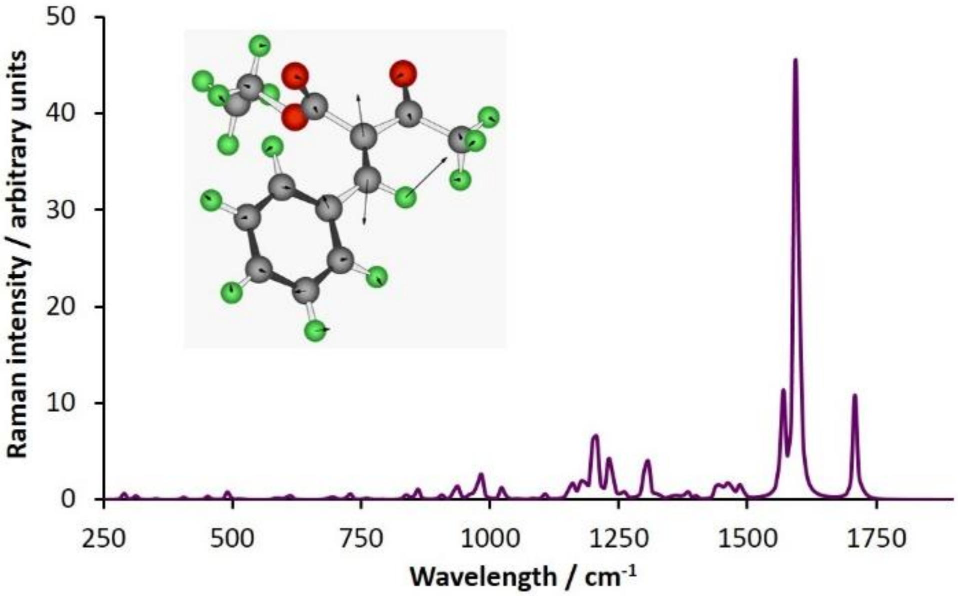

The Raman spectrum of **2a** generated using Gaussian 09 at the B3LYP/6-31g(d) level of theory. The inset molecule illustrates the complex stretching mode responsible for the signal calculated at 1600 cm¯1 (actual: 1598 cm^{-1}).

Claisen–Schmidt reaction

(*E*)-3-(4-Fluorophenyl)-3-phenylprop-2-en-1-one (3d):

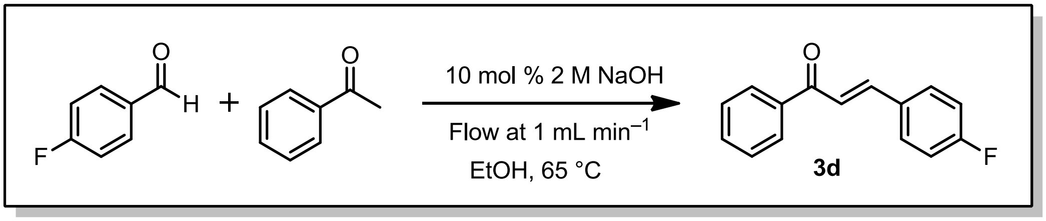

Into a 50 mL volumetric flask was added 4-fluorobenzaldehyde (1.551 g, 12.5 mmol, 1 equiv) and ethyl acetoacetate (1.637 g, 12.5 mmol, 1 equiv). Ethanol was added to bring the total volume to 50 mL (0.25 M) and the reagents were thoroughly mixed. An aliquot of this solution (10 mL) was transferred to a 20 mL vial equipped with a Teflon-coated stir bar. The flow reactor was readied using the equipment manufacturer's suggested start-up sequence. Ethanol was pumped at 0.5 mL/min to prime the system as the coil was heated to 65 °C. The back-pressure regulator was adjusted to 7 bar to prevent the solvent from boiling in the coil. After the heating coil, the product stream was intercepted by pumping acetone at 0.5 mL/min by means of a T-piece to ensure complete solubility of the product. The Raman probe was inserted into the box containing the flow cell and was properly focused. The background scan of the solvent system was taken that would be subtracted from all subsequent scans. 2 M NaOH (0.125 mL, 0.25 mmol) was injected all at once and after mixing for 15 s the reaction mixture was loaded into the reactor coil at a flow rate of 0.5 mL/min. Product collection was commenced immediately after this switch. After the reaction mixture had been completely loaded into the reactor, the reactor pump was set back to pumping ethanol. The Raman spectrometer was programmed to take continuous scans using the same parameters as the coumarin synthesis, above. After the product had been fully discharged from the flow cell, the scans were halted. The yellow solution was poured into a beaker containing ice (100 g) causing an immediate precipitation of the product. To ensure complete precipitation, the solution was stirred at 0 °C. The solid product was collected via vacuum filtration and washed with cold ethanol. The material was dried on top of the oven for 6 hours to yield (E)-3-(4-fluorophenyl)-3-phenylprop-2-en-1-one, **3d** (0.5421 g, 91%) as a pale yellow solid.

^{1}H NMR ($CDCl_3$, 400 MHz) δ ppm 7.11 (t, *J* = 8.68 Hz, 2 H), 7.46 (d, *J* = 15.89 Hz, 1 H), 7.49 - 7.55 (m, 2 H), 7.56 - 7.69 (m, 3 H), 7.78 (d, *J* = 15.65 Hz, 1 H), 8.02 (d, *J* = 7.34 Hz, 2 H).

^{13}C NMR ($CDCl_3$, 100 MHz) δ ppm 116.42 (d, $J_{C\text{-}C\text{-}F}$ = 22.01 Hz, CH), 122.09 (d, $J_{C\text{-}C\text{-}C\text{-}C\text{-}F}$ = 2.20 Hz, C), 128.76 (s, 10 C), 128.94 (s, 9 C), 130.62 (d, $J_{C\text{-}C\text{-}C\text{-}F}$ = 8.80 Hz, CH), 131.45 (d, $J_{C\text{-}C\text{-}C\text{-}C\text{-}C\text{-}F}$ = 3.67 Hz, CH), 133.12 (C), 138.43 (C), 143.78 (CH), 164.35 (d, $J_{C\text{-}F}$ = 250.89 Hz, C), 190.59 (C).

^{19}F NMR ($CDCl_3$, 377 MHz) δ ppm -113.59 - -111.32 (m, 1 F).

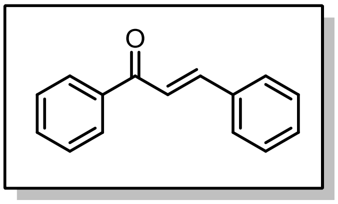

Chalcone (3a)
(90% GC conversion) was prepared according to the representative procedure for the synthesis of **3d** from benzaldehyde (0.2653 g, 2.5 mmol).

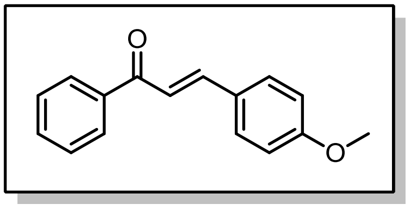

(*E*)-3-(4-methoxyphenyl)-1-phenylprop-2-en-1-one (3b)
(66% GC conversion) was prepared according to the representative procedure for the synthesis of **3d** from 4-methoxybenzaldehyde (0.3404 g, 2.5 mmol).

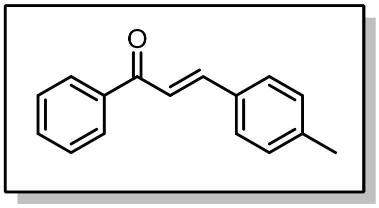

(*E*)-1-phenyl-3-(p-tolyl)prop-2-en-1-one (3c)
(84% GC conversion) was prepared according to the representative procedure for the synthesis of **3d** from 4-methylbenzaldehyde (0.3004 g, 2.5 mmol).

Biginelli reaction

Ethyl-6-methyl-2-oxo-4-phenyl-1,2,3,4-tetrahydropyrimidine-5-carboxylate (4a):

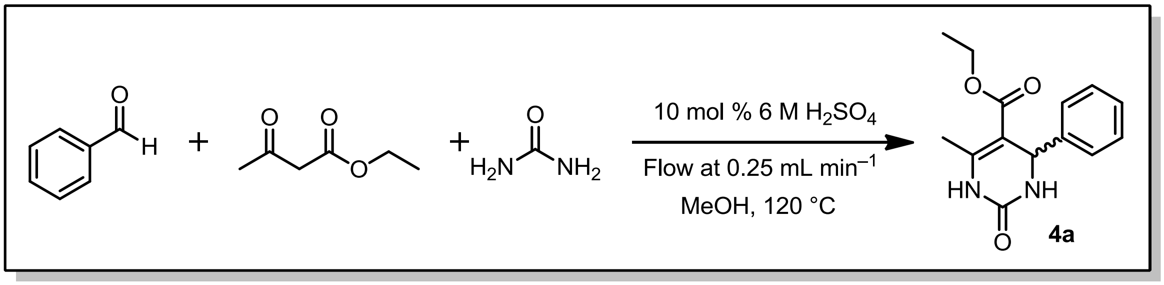

In a 50 mL volumetric flask was dissolved urea (3.003 g, 50 mmol, 1 equiv) in methanol (~30 mL). Into the flask was then added benzaldehyde (1.306 g, 50 mmol, 1 equiv), ethyl acetoacetate (6.507 g, 50 mmol, 1 equiv). Methanol was added to bring the total volume to 50 mL (1 M) and the reagents were thoroughly mixed. An aliquot of this solution (10 mL) was transferred to a 20 mL vial equipped with a Teflon-coated stir bar. The flow reactor was readied using the equipment manufacturer's suggested start-up sequence. Methanol was pumped at 0.25 mL min^{-1} to prime the system as the coil was heated to 120 °C. The back-pressure regulator was adjusted to 7 bar to prevent the solvent from boiling in the coil. After the heating coil, the product stream was intercepted by pumping DMF (0.25 mL/min) by means of a T-piece to ensure complete solubility of the product. The Raman spectrometer was set to acquire a spectrum every 25 s, with 20 s integration time, boxcar = 3, and average = 1. When the flow unit was ready, 6 M H_2SO_4 (0.2 mL, 0.1 equiv) was injected all at once and after mixing for 15 s the reaction mixture was loaded into the reactor coil at a flow rate of 0.25 mL/min. After the reaction mixture had been completely loaded into the reactor, methanol was again pumped through the coil at 0.25 mL/ min. Product collection was commenced immediately after this switch. After the reaction mixture had been completely loaded into the reactor, the reactor pump was set back to pumping methanol. After the product had been fully discharged from the flow cell, the scans were halted. The reaction mixture was transferred to a separatory funnel, diluted with diethyl ether and sat. $NaHCO_3$ (100 mL) and water (100 mL) added. The layers were separated and the aqueous layer extracted with ether (3 × 100 mL). The combined organics were washed with brine (2 × 100 mL) and dried over Na_2SO_4. The solvent was removed *in vacuo* by rotary evaporation affording the crude product. The resulting solid was transferred to a filter funnel, washed with cold methanol and air dried to afford **4a** (2.03 g, 78%) as a fluffy white solid.

^{1}H NMR (DMSO-d_6, 500 MHz) δ ppm 1.18 (s., 3 H), 2.34 (s., 3 H), 3.67 - 4.60 (m, 2 H), 5.24 (s., 1 H), 7.34 (s., 5 H), 7.80 (s., 1 H), 7.74 (s, 1 H), 9.26 (s., 1 H).

^{13}C NMR (d_6-DMSO-d_6, 125 MHz) δ ppm 14.5 (CH_3), 18.2 (CH_3), 54.4 (CH), 59.6 (CH_2), 99.7 (C), 126.7 (CH), 127.7 (CH), 128.8 (CH), 145.3 (C), 148.8 (C), 152.6 (C), 165.8 (C).

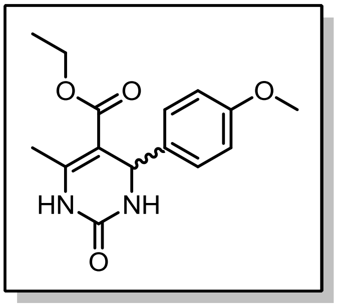

5-Ethoxycarbonyl-6-methyl-4-(4-methoxyphenyl)-3,4 dihydropyrimidin-2(1*H*)-one (4b)

(85% GC conversion) was prepared according to the representative procedure for the synthesis of **4a** from 4-methoxybenzaldehyde (1.3615 g, 10 mmol).

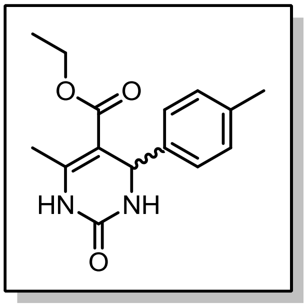

5-Ethoxycarbonyl-6-methyl-4-(4-methylphenyl)-3,4 dihydropyrimidin-2(1*H*)-one (4c)

(87% GC conversion) was prepared according to the representative procedure for the synthesis of **4a** from 4-methylbenzaldehyde (1.2015 g, 10 mmol).

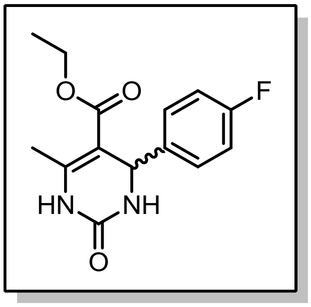

5-Ethoxycarbonyl-6-methyl-4-(4-fluorophenyl)-3,4 dihydropyrimidin-2(1*H*)-one (4d)

(91% GC conversion) was prepared according to the representative procedure for the synthesis of **4a** from 4-fluorobenzaldehyde (1.2411 g, 10 mmol).

Catesian coordinates of all stationary points

3-Acetylcoumarin (1a):

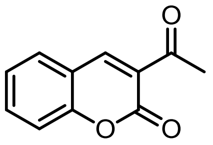

ATOM	X	Y	Z
0 1			
C	-2.67580800	1.19509900	0.00008900
C	-1.37232100	0.69429900	-0.00002200
C	-1.12088200	-0.69189100	-0.00005300
C	-2.21731600	-1.57893300	0.00003200
C	-3.51389300	-1.09009800	0.00016000
C	-3.73793300	0.29757500	0.00018900
H	-2.83054200	2.26873400	0.00016100
H	-2.02599800	-2.64865700	-0.00001900
H	-4.35586300	-1.77524000	0.00020200
H	-4.75529800	0.67831400	0.00031400
C	0.24672500	-1.10813100	-0.00004200
H	0.49416300	-2.16701900	-0.00007000
O	-0.34929800	1.59023300	-0.00000800
C	1.00236300	1.22358800	-0.00018300
C	1.28001200	-0.21785400	-0.00001300
O	1.80458000	2.12736700	-0.00074800
C	2.68341400	-0.77456300	0.00006400
O	2.83153300	-1.98831500	-0.00075600
C	3.87377100	0.15555600	0.00108300
H	3.85510200	0.81576600	-0.87191200
H	3.85337100	0.81653700	0.87344400
H	4.78173700	-0.45060500	0.00215200

(*Z*)-Ethyl 2-benzylidene-3-oxobutanoate (2a):

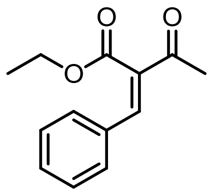

Coordinates (from last standard orientation):

ATOM	X	Y	Z
0 1			
C	-0.37767600	0.86162900	0.51581700
C	-0.01168800	0.07913500	1.61285900
C	1.33474100	-0.12498900	1.89949800
C	2.34643900	0.42705700	1.08793300
C	1.96123400	1.22787200	-0.00596400
C	0.61268800	1.43816300	-0.28327900
H	-1.42742500	1.03111200	0.29205300
H	-0.77359600	-0.36535300	2.24723100
H	1.61624500	-0.73132300	2.75745800
H	2.71513200	1.71118800	-0.61488000
H	0.33366800	2.06575500	-1.12530200
C	3.73555600	0.14233500	1.46197000
H	3.83535900	-0.19447400	2.49229800
C	4.88211800	0.18486000	0.74559300
C	4.98337600	0.57005400	-0.70885900
C	6.20889700	-0.15315500	1.34104200
O	7.21675700	-0.05783700	0.65445000
O	4.94476500	1.70626700	-1.13321200
O	5.14032900	-0.51908800	-1.47587700
C	5.36607100	-0.27522400	-2.88646300
H	4.49139300	0.23939900	-3.29753500
H	6.22652000	0.39266500	-2.98859600
C	5.60183500	-1.62036500	-3.54596400
H	5.77675300	-1.48163100	-4.61852000
H	6.47659300	-2.11635600	-3.11413400
H	4.73448900	-2.27642500	-3.42004800
C	6.30035200	-0.59937800	2.79254400
H	5.93892500	0.18019900	3.47303100
H	5.70469100	-1.50149600	2.97347000
H	7.34764700	-0.80974000	3.01580900

(*E*)-3-(4-Fluorophenyl)-3-phenylprop-2-en-1-one (3d):

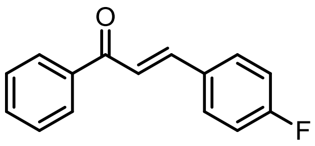

Coordinates (from last standard orientation):

ATOM	X	Y	Z
0 1			
C	0.10925400	0.03032200	-0.19769200
C	-0.07675900	0.24069900	1.16714900
C	1.03336300	0.23966000	2.00224400
C	2.33139500	0.03089600	1.49286200
C	2.47061500	-0.17777100	0.10714700
C	1.36942900	-0.17980900	-0.74440100
H	-1.07878800	0.40080100	1.55127700
H	0.88864400	0.40314200	3.06563900
H	3.46288500	-0.34067800	-0.30504800
H	1.47286900	-0.33992200	-1.81238900
F	-0.96602000	0.03102300	-1.00861500
C	3.53110700	0.02076300	2.32559200
H	4.47211800	-0.15071000	1.80430100
C	3.62230600	0.19875900	3.65932800
H	2.74072800	0.38842400	4.26195000
C	4.94975500	0.16846600	4.32218000
C	5.02650000	0.29958800	5.81687900
C	3.91530500	0.19072600	6.66747600
C	6.29251100	0.52363400	6.38276200
C	4.06682100	0.30880200	8.04926300
H	2.92747600	-0.00808000	6.26480000
C	6.44250500	0.65071400	7.75976900
H	7.14439000	0.59382300	5.71466300
C	5.32828600	0.54399900	8.59770100
H	3.19931400	0.21526800	8.69690900
H	7.42684100	0.83101100	8.18345200
H	5.44402500	0.64094600	9.67400600
O	5.98309500	0.05270000	3.66511900

Ethyl-6-methyl-2-oxo-4-phenyl-1,2,3,4-tetrahydropyrimidine-5-carboxylate (4a):

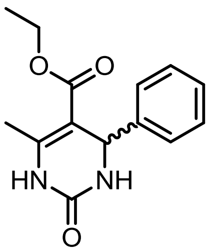

Coordinates (from last standard orientation):

ATOM	X	Y	Z
0 1			
C	-1.35407100	2.57048200	-0.39022700
C	-0.72642100	0.24466900	-1.04545600
C	0.59063100	0.47613500	-0.30388600
C	0.70847300	1.55472000	0.52130800
H	-0.47567800	-0.17992100	-2.02101500
O	-2.11254200	3.52860600	-0.40157500
N	-0.33360900	2.46568100	0.56743300
H	-0.26143100	3.27811200	1.16399700
N	-1.36487900	1.54181300	-1.28714500
H	-2.16398300	1.56133800	-1.90790500
C	-1.64591600	-0.75523200	-0.32650900
C	-1.47446100	-2.12953600	-0.54839400
C	-2.65207800	-0.33633400	0.55361000
C	-2.28232900	-3.06095300	0.10370200
H	-0.69501100	-2.46212100	-1.22661600
C	-3.46070300	-1.26972400	1.20530500
H	-2.82552000	0.72153100	0.72614400
C	-3.27827200	-2.63502400	0.98460700
H	-2.13699800	-4.12195700	-0.08310800
H	-4.23866800	-0.92535300	1.88193100
H	-3.91111800	-3.36044200	1.48911500
C	1.87444800	1.91085900	1.40983200
H	2.19414600	1.05530700	2.00514200
H	2.73669400	2.22584700	0.81384500
H	1.60404400	2.73056200	2.08356100
C	1.64047200	-0.50607600	-0.59521700
O	2.79405600	-0.33165900	0.10127200
O	1.51313100	-1.40663200	-1.41317700
C	3.84376300	-1.28177900	-0.18798800
H	4.08323700	-1.23088500	-1.25482500
H	3.47425900	-2.29135400	0.01763400
C	5.03630400	-0.92498700	0.67997600
H	4.78033100	-0.98123000	1.74315600
H	5.85655500	-1.62519800	0.48779300
H	5.39235500	0.08747600	0.46300100

^{1}H-NMR spectra of synthesized compounds

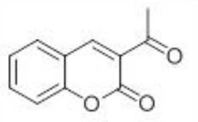

1a

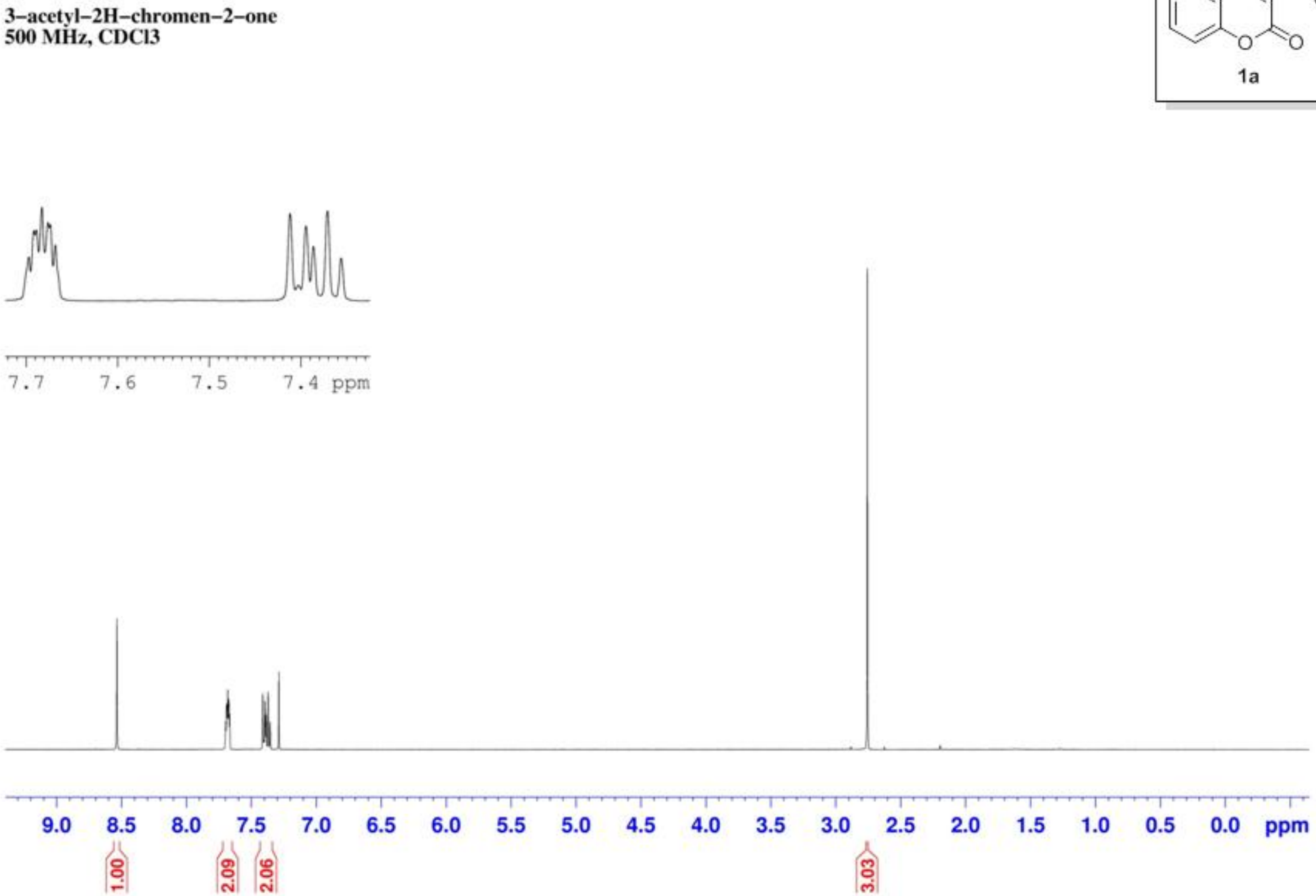

(Z)–ethyl 2–benzylidene–3–oxobutanoate
400 MHz, CDCl3

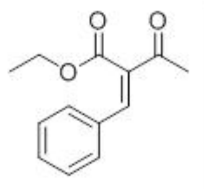

2a

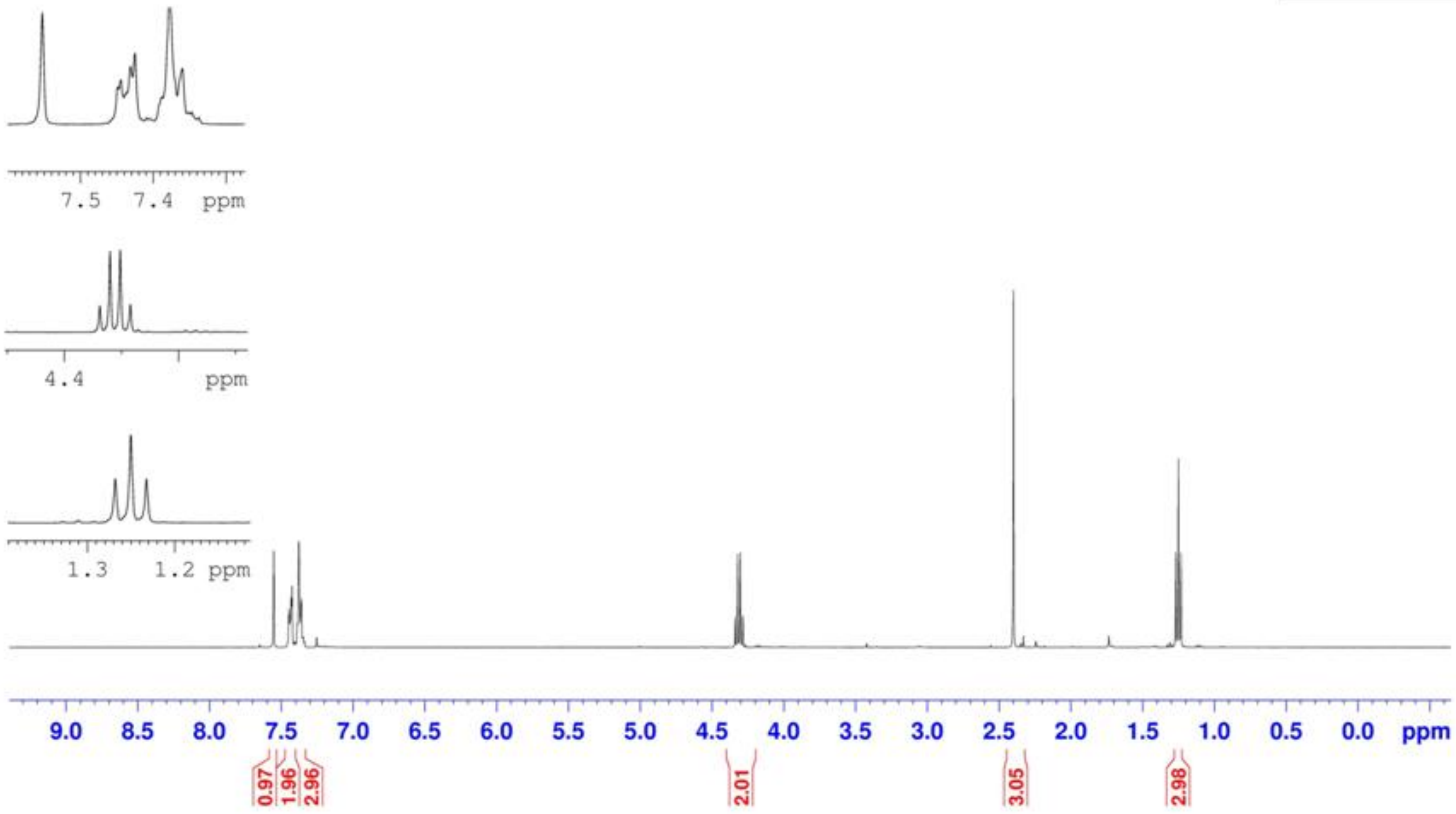

(E)–3–(4–fluorophenyl)–1–phenylprop–2–en–1–one
400 MHz, CDCl3

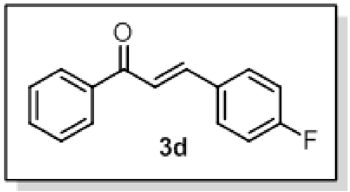

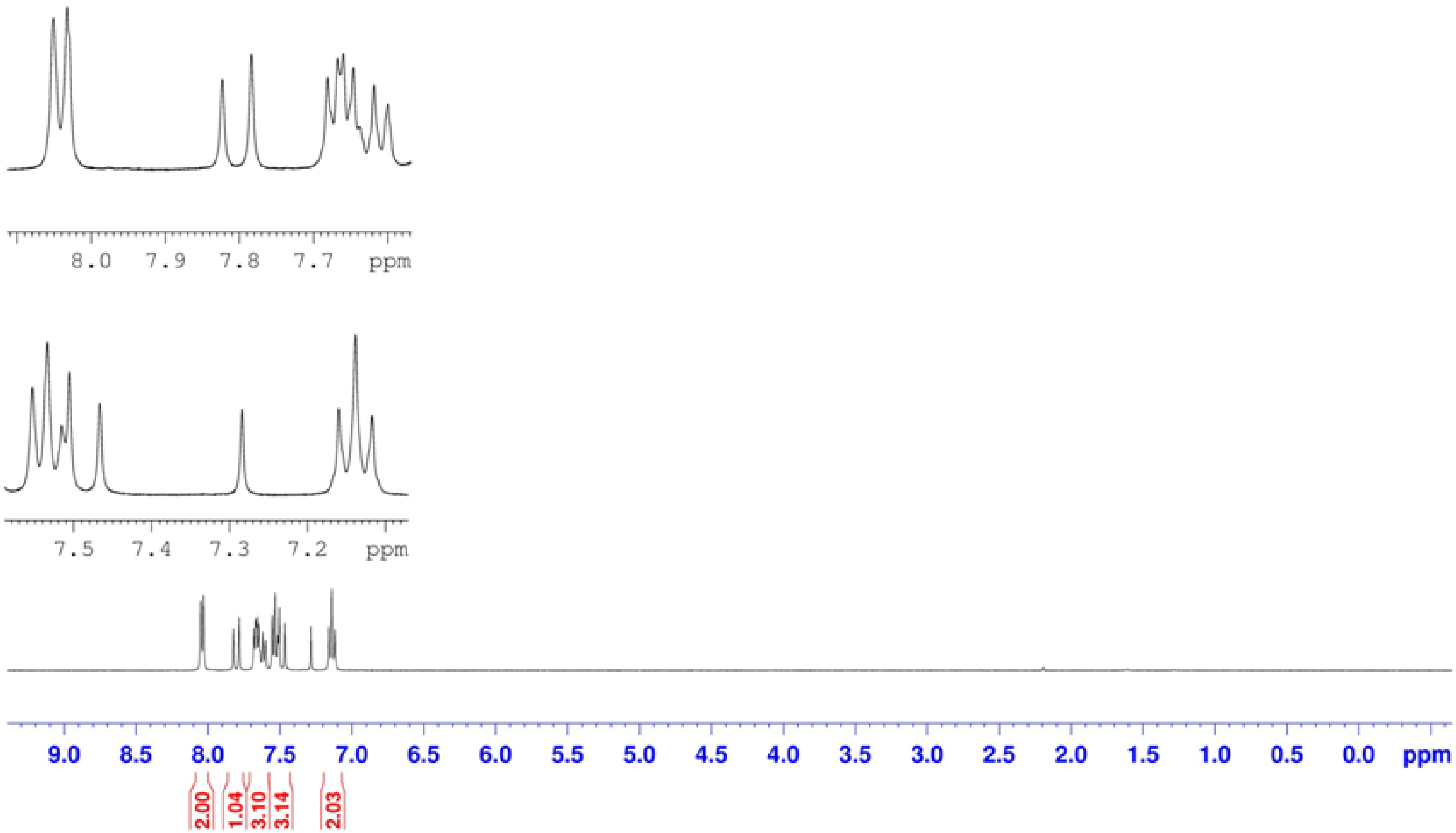

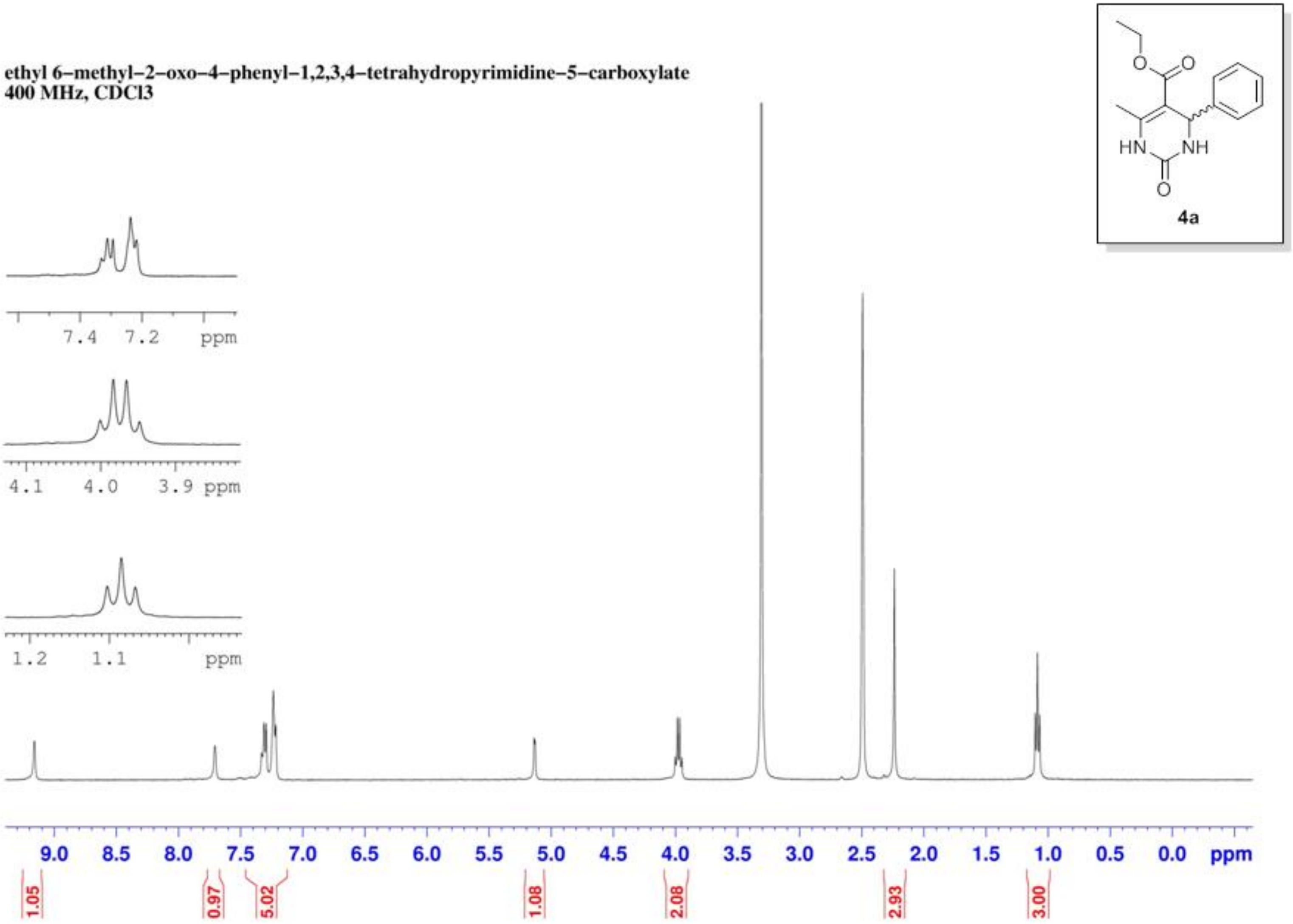
ethyl 6–methyl–2–oxo–4–phenyl–1,2,3,4–tetrahydropyrimidine–5–carboxylate
400 MHz, CDCl3
HN
NH
O
4a
7.4 7.2 ppm
4.1 4.0 3.9 ppm
1.2 1.1 ppm
9.0 8.5 8.0 7.5 7.0 6.5 6.0 5.5 5.0 4.5 4.0 3.5 3.0 2.5 2.0 1.5 1.0 0.5 0.0 ppm
1.05
0.97
5.02
1.08
2.08
2.93
3.00

^{13}C-NMR spectra of synthesized compounds

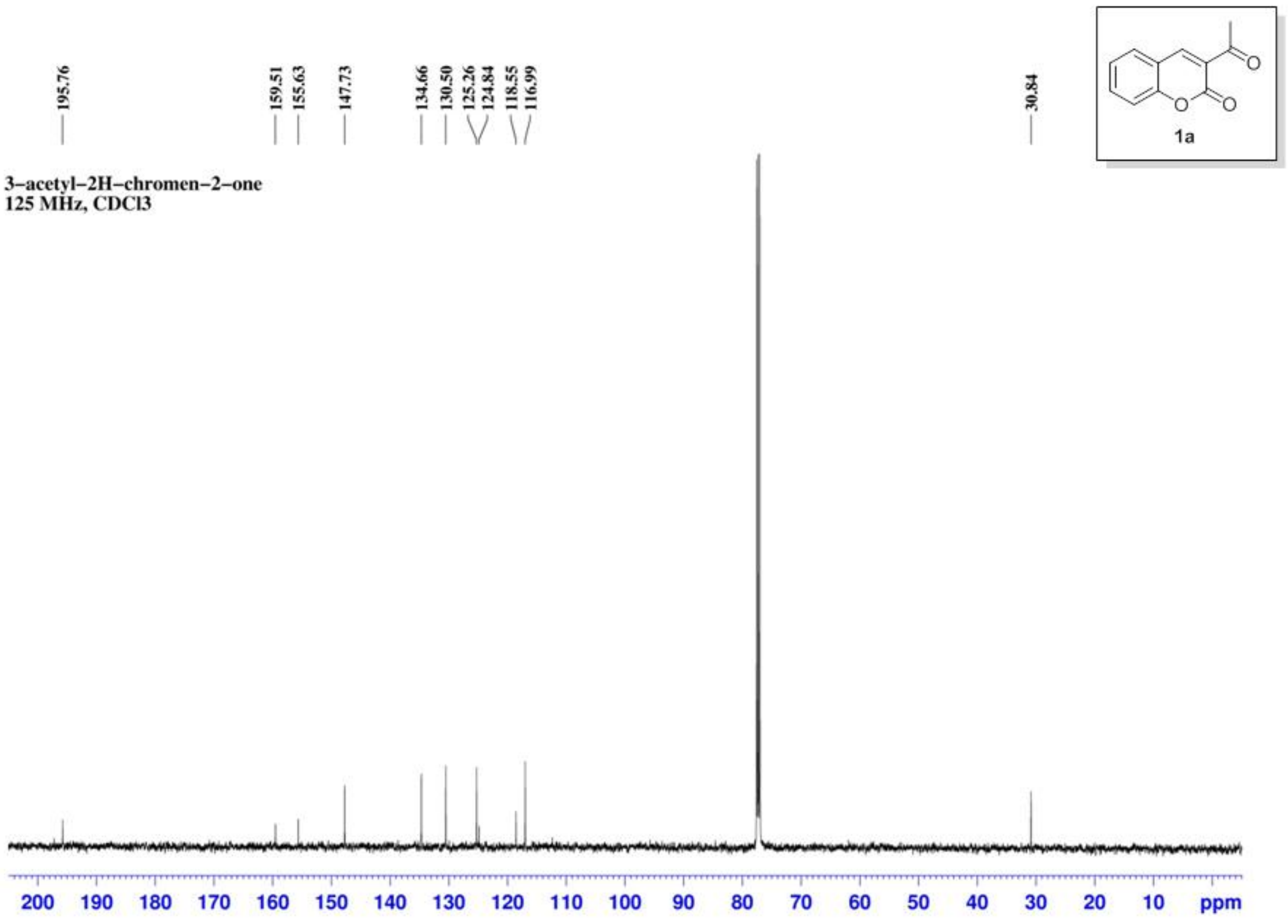

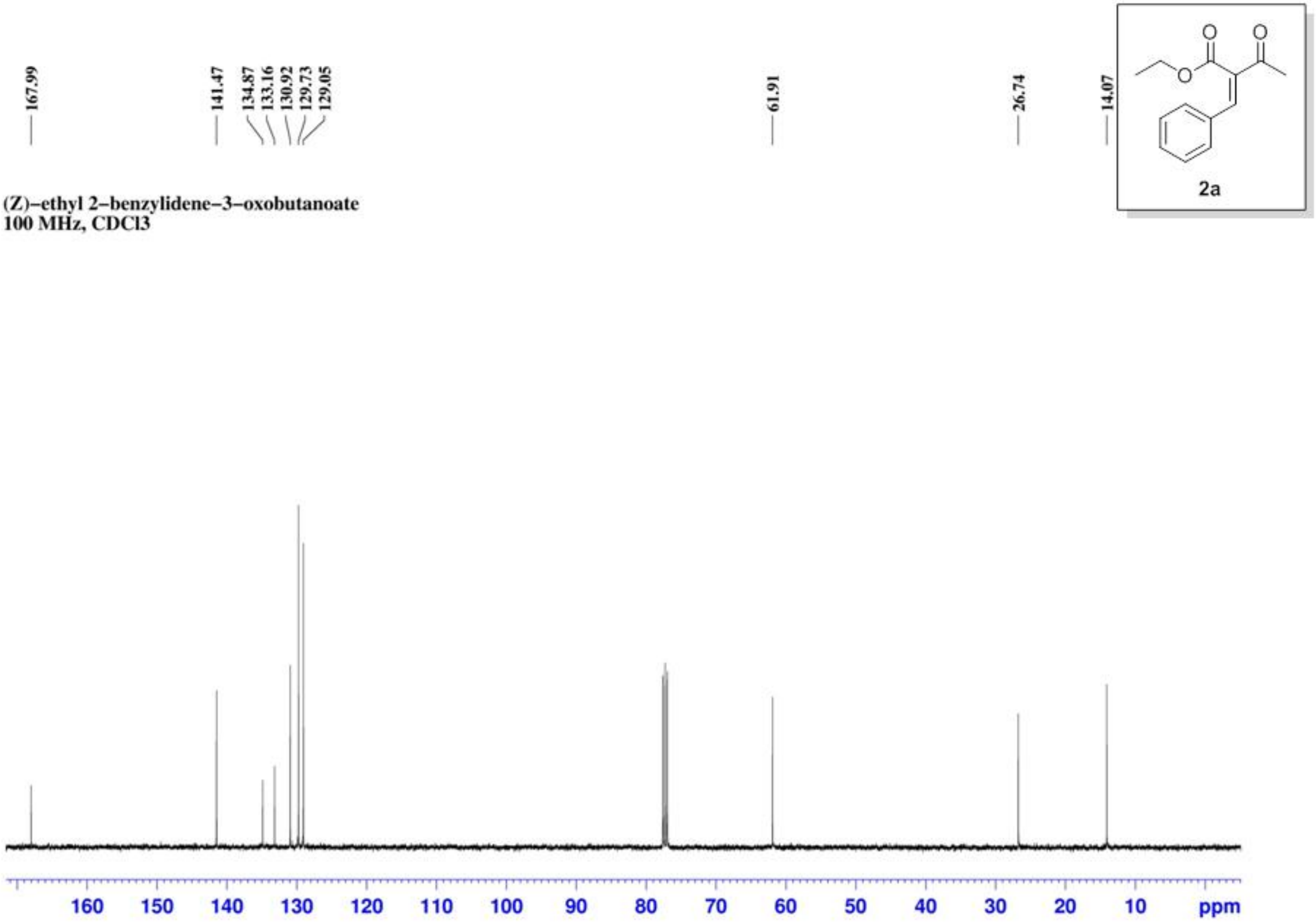
167.99
141.47
134.87
133.16
130.92
129.73
129.05
61.91
26.74
14.07
(Z)−ethyl 2−benzylidene−3−oxobutanoate
100 MHz, CDCl3
2a
160 150 140 130 120 110 100 90 80 70 60 50 40 30 20 10 ppm

(E)-3-(4-fluorophenyl)-1-phenylprop-2-en-1-one
100 MHz, CDCl3

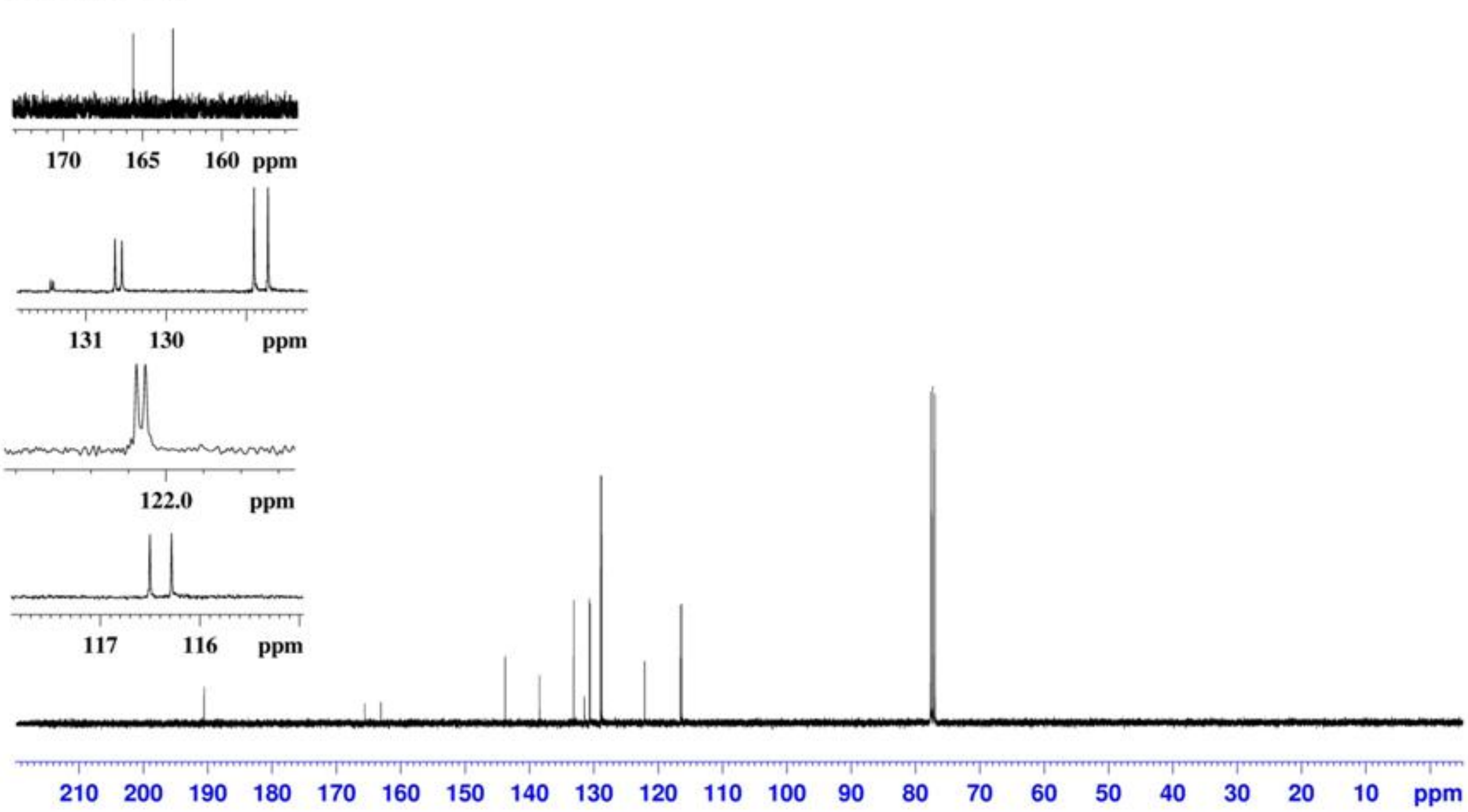

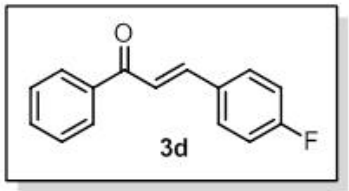

ethyl 6–methyl–2–oxo–4–phenyl–1,2,3,4–tetrahydropyrimidine–5–carboxylate
125 MHz, DMSO

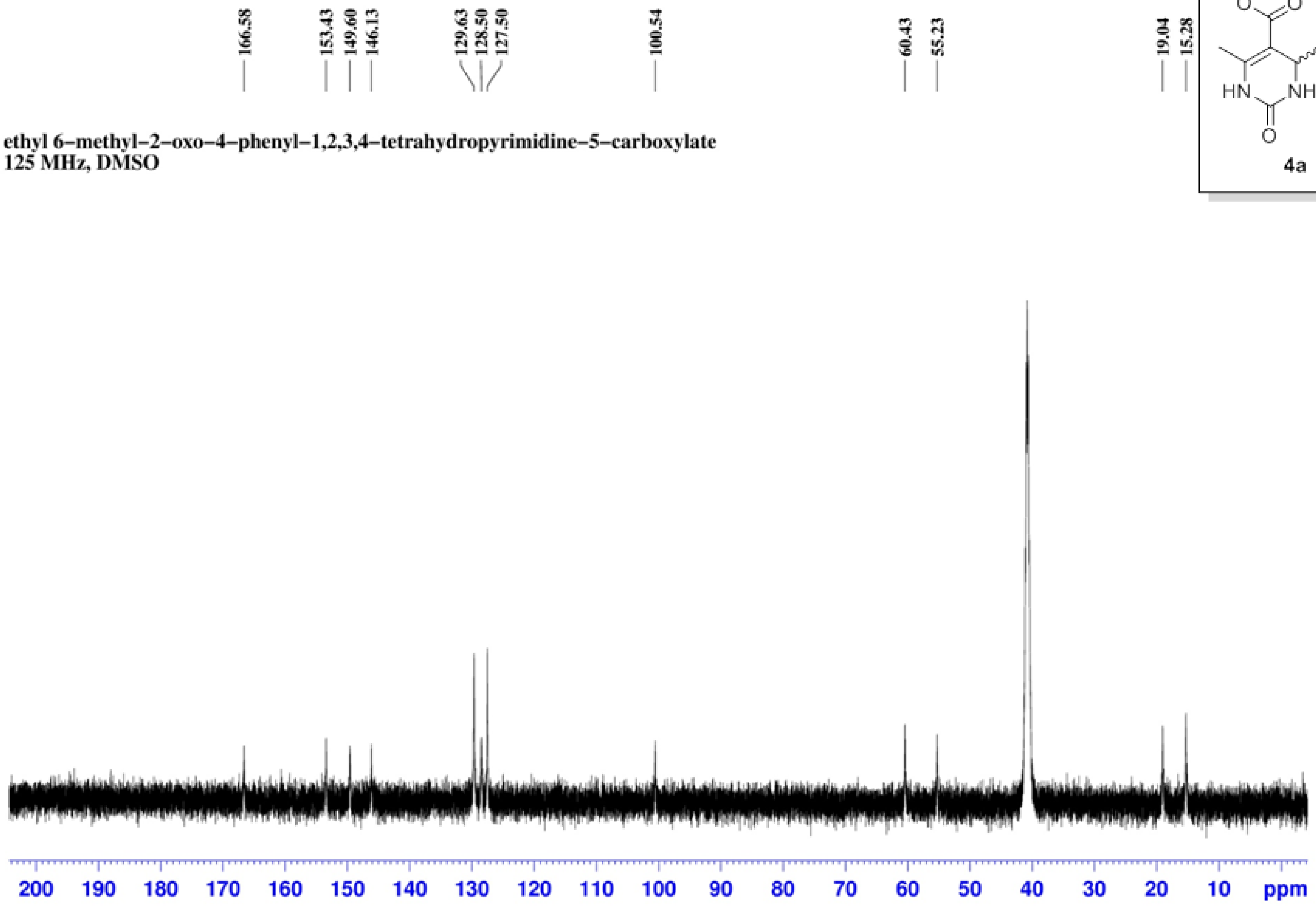

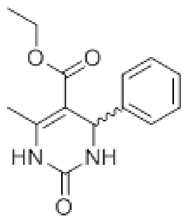

4a

^{19}F-NMR spectra of synthesized compounds

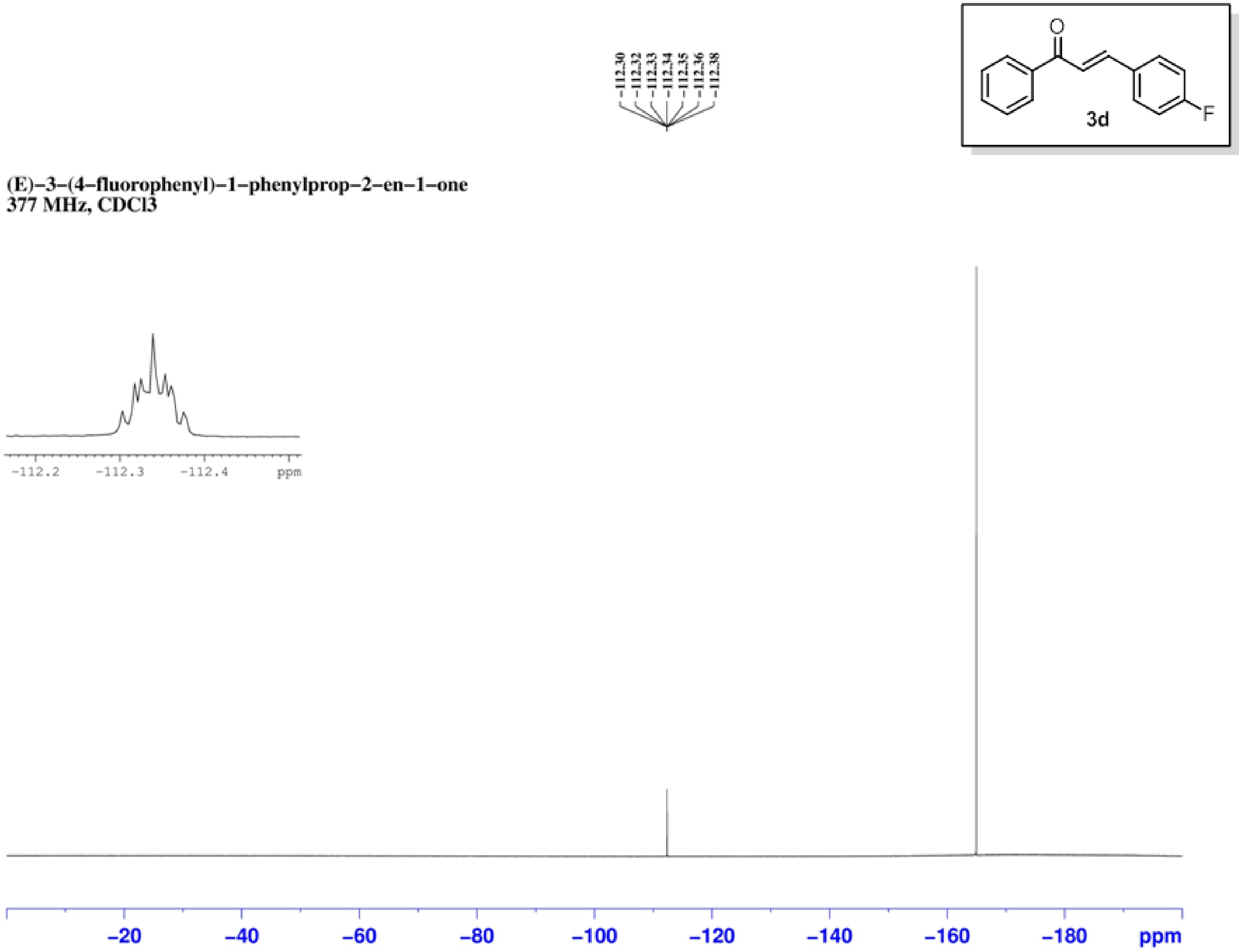

Supplement: File 1 — NMR spectra of the isolated products (1, 2a, 3d, 4a), further experimental information, and pictures of the Raman interface and Cartesian coordinates of the stationary points. [file Beilstein_J_Org_Chem-09-1843-s001.pdf]
